# Integration of bio-inspired lanthanide-transition metal cluster and P-doped carbon nitride for efficient photocatalytic overall water splitting

**DOI:** 10.1093/nsr/nwaa234

**Published:** 2020-09-14

**Authors:** Rong Chen, Gui-Lin Zhuang, Zhi-Ye Wang, Yi-Jing Gao, Zhe Li, Cheng Wang, Yang Zhou, Ming-Hao Du, Suyuan Zeng, La-Sheng Long, Xiang-Jian Kong, Lan-Sun Zheng

**Affiliations:** Collaborative Innovation Center of Chemistry for Energy Materials, State Key Laboratory of Physical Chemistry of Solid Surface and Department of Chemistry, College of Chemistry and Chemical Engineering, Xiamen University, Xiamen 361005, China; College of Chemical Engineering, Zhejiang University of Technology, Hangzhou 310032, China; Collaborative Innovation Center of Chemistry for Energy Materials, State Key Laboratory of Physical Chemistry of Solid Surface and Department of Chemistry, College of Chemistry and Chemical Engineering, Xiamen University, Xiamen 361005, China; College of Chemical Engineering, Zhejiang University of Technology, Hangzhou 310032, China; Collaborative Innovation Center of Chemistry for Energy Materials, State Key Laboratory of Physical Chemistry of Solid Surface and Department of Chemistry, College of Chemistry and Chemical Engineering, Xiamen University, Xiamen 361005, China; Collaborative Innovation Center of Chemistry for Energy Materials, State Key Laboratory of Physical Chemistry of Solid Surface and Department of Chemistry, College of Chemistry and Chemical Engineering, Xiamen University, Xiamen 361005, China; Collaborative Innovation Center of Chemistry for Energy Materials, State Key Laboratory of Physical Chemistry of Solid Surface and Department of Chemistry, College of Chemistry and Chemical Engineering, Xiamen University, Xiamen 361005, China; Collaborative Innovation Center of Chemistry for Energy Materials, State Key Laboratory of Physical Chemistry of Solid Surface and Department of Chemistry, College of Chemistry and Chemical Engineering, Xiamen University, Xiamen 361005, China; College of Chemistry and Chemical Engineering, Liaocheng University, Liaocheng 252059, China; Collaborative Innovation Center of Chemistry for Energy Materials, State Key Laboratory of Physical Chemistry of Solid Surface and Department of Chemistry, College of Chemistry and Chemical Engineering, Xiamen University, Xiamen 361005, China; Collaborative Innovation Center of Chemistry for Energy Materials, State Key Laboratory of Physical Chemistry of Solid Surface and Department of Chemistry, College of Chemistry and Chemical Engineering, Xiamen University, Xiamen 361005, China; Collaborative Innovation Center of Chemistry for Energy Materials, State Key Laboratory of Physical Chemistry of Solid Surface and Department of Chemistry, College of Chemistry and Chemical Engineering, Xiamen University, Xiamen 361005, China

**Keywords:** bio-inspired, lanthanide-transition metal cluster, photocatalytic overall water splitting, synergistic effects, oxygen-evolving center

## Abstract

Photosynthesis in nature uses the Mn_4_CaO_5_ cluster as the oxygen-evolving center to catalyze the water oxidation efficiently in photosystem II. Herein, we demonstrate bio-inspired heterometallic LnCo_3_ (Ln = Nd, Eu and Ce) clusters, which can be viewed as synthetic analogs of the CaMn_4_O_5_ cluster. Anchoring LnCo_3_ on phosphorus-doped graphitic carbon nitrides (PCN) shows efficient overall water splitting without any sacrificial reagents. The NdCo_3_/PCN-c photocatalyst exhibits excellent water splitting activity and a quantum efficiency of 2.0% at 350 nm. Ultrafast transient absorption spectroscopy revealed the transfer of a photoexcited electron and hole into the PCN and LnCo_3_ for hydrogen and oxygen evolution reactions, respectively. A density functional theory (DFT) calculation showed the cooperative water activation on lanthanide and O−O bond formation on transition metal for water oxidation. This work not only prepares a synthetic model of a bio-inspired oxygen-evolving center but also provides an effective strategy to realize light-driven overall water splitting.

## Teaser

This work demonstrates an efficient overall water splitting catalysts via anchoring bio-inspired heterometallic clusters LnCo^II^Co^III^_2_ on phosphorus-doped graphitic carbon nitrides.

## INTRODUCTION

Green plants use a cubane-type {CaMn_4_O_5_} cluster for catalyzing the water oxidation reaction in the oxygen evolution center (OEC) of photosystem II (PSII) [1–4], which is a critical half reaction for converting sunlight energy into chemical energies stored in ATP and NADPH. Synergistic effect among the multi-metal centers of the OEC plays a key role for the high catalytic activity of PSII [5,6]. Ca^2+^ serves to adsorb and activate the H_2_O molecule, while Mn with variable oxidation states in the cluster provides the oxidative equivalents. Nature chooses Ca and Mn as the elements to build the cluster, partly because of the availability of the two elements in the environment. To mimic nature, we can use any elements available to us. Lanthanide ions can be a better Lewis acid than Ca^2+^ and Co is found to be a common element in water oxidation catalysts. As a result, a lanthanide-cobalt cluster may be a good biomimetic water oxidation catalyst [7]. Mimicking natural photosynthesis, light-driven overall water splitting to produce H_2_ and O_2_ including both the hydrogen evolution reaction (HER) and oxygen evolution reaction (OER) is a promising pathway for artificial conversion and storage of solar energy [8–10]. The OER side is usually the rate-determining step. Inspired by the structure model of PSII, some interesting heterometallic cubane-like clusters have been designed and synthesized to act as bio-inspired water oxidation catalysts [11–16]. Sacrificial agents are used to test the performance of these catalysts. However, the natural OEC functions in an integrated system to optimize overall efficiency of a sequence of events including charge separation, charge transfer and catalytic reaction, which diminishes charge recombination. We envisioned that the synthetic biomimetic OECs should also be studied in an integrated system to reveal their true potentials and provide a better understanding of the synergistic effect in catalysis on an atomic level.

Various approaches have been put forward to build integrated systems for improving the separation and transportation of photo-generated electron-hole pairs [17–21]. For example, close connection between the catalytic center and the photosensitive center can effectively reduce charge recombination rates [22–25]. Assembling bio-inspired OECs on the surface of two-dimensional (2D) layered semiconductor materials may enhance photocatalytic overall water splitting, which uses the connection between the OEC and the 2D materials as a junction to improve charge separation and fine tune surface electronic structure. Herein, we synthesized a heterometallic cluster LnCo^II^Co^III^_2_ (LnCo_3_), which is a structural analog to the CaMn_4_O_5_ of PSII. By anchoring the bio-inspired LnCo_3_ as the OEC on phosphorus-doped (P-doped) graphitic carbon nitrides (PCN), we realized light-driven spontaneous overall water splitting to efficiently produce O_2_ and H_2_. The NdCo_3_/PCN-c exhibited remarkable water-splitting activity with a high H_2_ production rate of ∼297.7 μmol h^−1^ g^−1^ and O_2_ evolution rate of 148.9 μmol h^−1^ g^−1^ under light irradiation. The photoexcited electron-hole pairs would be easily dissociated and transferred into the PCN and LnCo_3_ to participate in HER and OER, respectively.

## RESULTS AND DISCUSSION

**Figure 1.**
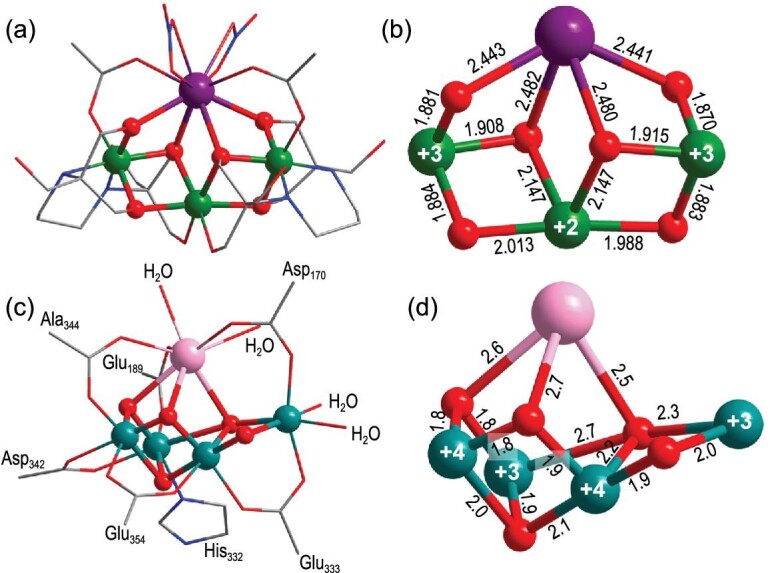
Crystal structures of the synthetic NdCo_3_ cluster and the native CaMn_4_ of PSII. (a) Crystal structure of NdCo_3_. (b) Structure of [NdCo^II^Co^III^_2_] core. (c) Structure of the native CaMn_4_O_5_ of PSII. (d) CaMn^III^_2_Mn^IV^_2_O_5_ core in PSII [1]. Nd, Co, Mn, Ca, O, N and C are shown in purple, green, teal, pink, red, blue and gray, respectively. For clarity, all H atoms are omitted.

LnCo_3_ was synthesized by reacting Ln(NO_3_)_3_, Co(Ac)_2_ and bis-tris-propane (btp) in methanol solution. The LnCo_3_s are isostructural. Here we only describe the structure of NdCo_3_ in detail. Single X-crystal structural analysis showed that NdCo_3_ crystallizes in monoclinic P2(1)/n space group and contains one [NdCo^II^Co^III^_2_(btp-3H)_2_(Ac)_2_(NO_3_)_2_]^+^ cation core, one nitrate ion and two guest water molecules. In the metal core, each Co^3+^ is chelated by two N and three O atoms from one deprotonated btp-3H ligand, to form one stable [Co^III^(btp-3H)] unit (Supplementary Fig. 1). One Co^2+^ and one Nd^3+^ ion connect two [Co^III^(btp-3H)] units by coordinating to the six bridging-O and two bridging-COO^−^ from two btp-3H ligands, generating the heterometallic tetranuclear structure [NdCo^II^Co^III^_2_(btp-3H)_2_]^5+^ (Fig. 1a). In addition, two Ac^−^ ligands bridge the Nd^3+^ and its neighboring Co^3+^. There are two NO_3_^−^ anions coordinated to one Nd^3+^ in chelated bidentate (μ_2_) mode. The adjacent Nd^3+^, Co^2+^, Co^3+^ and three bridging O’s from one btp-3H ligand form a defective cubane [NdCo^II^Co^III^O_3_] motif. Two [NdCo^II^Co^III^O_3_] connect by sharing faces, resulting in [NdCo^II^Co^III^_2_] core (Fig. 1b). The distances of Co^II^-O range from 1.988 to 2.147 Å, and the distances of Co^III^-O/Co^III^-N range from 1.870 to 1.966 Å, both of which are consistent with the reported values of other cobalt structures [26–28]. According to the theoretical calculation formula of valence bond, BVS = exp((R_0_−R)/B), the states of the metal ions and the pronation states of each oxygen and nitrogen atom of the organic ligand were calculated. As shown in Supplementary Tables 11 and 12, the calculated results show that the oxidation states of all the lanthanide ions are +3 and the oxidation states of Co1 and Co2 are +2 and +3; the O7, O9 and O10 in btp-3H ligands show the O^2−^ state, while the O6, O5 and O8 atoms exhibit OH^−^ states.

Interestingly, the heterometallic cluster [NdCo^II^Co^III^_2_] mimics the structure of CaMn_4_O_5_ of PSII. Considering the monotonic change in radius and chemical properties of the lanthanides, it was an attractive choice for investigating the physical characteristics of the clusters [29]. In addition, due to the similarities in ionic radii and high coordination numbers of lanthanide ions and Ca^2+^, they can be exchanged in biological systems [30,31]. As shown in Fig. 1, topologically the NdCo_3_ cluster can be viewed as the CaMn_4_O_5_ missing one metal vertex from the cubane and adding one bridging-O atom between Nd^3+^ and Co^3+^. In addition, the coordination mode of bridging-O in the NdCo_3_ cluster is also very similar to that in CaMn_4_O_5_ of PSII, except that the five bridging-O atoms are O^2−^ in biological CaMn_4_O_5_-cluster, while six bridging-O atoms come from the −OH groups of two btp-3H ligands in NdCo_3_. Notably, the mixed oxidation states of the cobalt ions (+2 and +3) in the NdCo_3_ cluster are similar to the mixed oxidation states of manganese ions in CaMn_4_ (+3 and +4), suggesting that the NdCo_3_ cluster can be viewed as a synthetic model of the OEC [32–35]. Compared with the CaMn_4_O_5_, the NdCo_3_ cluster shows high stability because of the presence of a chelating btp-3H ligand. High-resolution electro-spray ionization mass spectrometry (HRESI-MS) of NdCo_3_ in methanol shows main peaks in the range of 1242 to 1252, which corresponds to the {[NdCo_3_(btp-3H)_2_(Ac)_2_(NO_3_)_2_](NO_3_)_2_}^−^ and the dimer structure {[NdCo_3_(btp-3H)_2_(Ac)_2_(NO_3_)_2_](NO_3_)_2_}_2_^2−^ (Supplementary Fig. 3). This result indicates that the NdCo_3_ cluster remains intact in methanol solution. The EuCo_3_ and CeCo_3_ clusters show the same crystal structure as NdCo_3_ (Supplementary Fig. 4).

**Figure 2.**
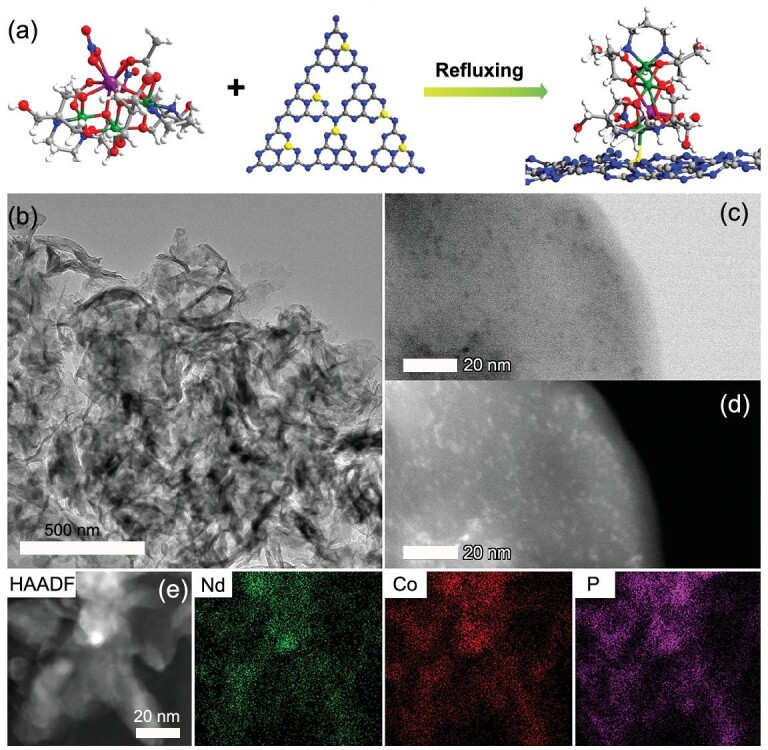
The synthetic schematic diagram process of NdCo_3_/PCN and electron microscopy of NdCo_3_/PCN-c. (a) Nd, Co, O, N, C and P are shown in purple, green, red, blue, gray and yellow, respectively. (b) TEM image of NdCo_3_/PCN-c. (c) and (d) Representative HAADF-STEM images of NdCo_3_/PCN-c. (e) Elemental mappings of Nd, Co and P. Nd: green; Co: red; P: purple.

Based on the stability of the cluster in methanol solution, anchoring NdCo_3_ clusters on PCN was prepared as shown in Fig. 2a. Forty-five milligrams of prepared PCN was dispersed in methanol solution (3 mg/mL) with sonication and then transferred to the flask with stirring. One milliliter of NdCo_3_ methanol solution (1, 2, 3, 5 and 7 mg mL^−1^) was dripped into the PCN suspension and refluxed for 12 h. The resultant precipitates were collected by filtration and dried at 70^o^C overnight, resulting in NdCo_3_/PCN samples with different loading amounts of the clusters (as measured by inductively coupled plasma mass spectrometry [ICP-MS]), as denoted: NdCo_3_/PCN-a (0.36 wt%), NdCo_3_/PCN-b (0.61 wt%), NdCo_3_/PCN-c (1.05 wt%), NdCo_3_/PCN-d (1.55 wt%) and NdCo_3_/PCN-e (2.03 wt%) (Supplementary Table 6).

Transmission electron microscopy (TEM) shows that the obtained NdCo_3_/PCN exhibits the morphology of nanosheets (Fig. 2b). To determine the distribution of the NdCo_3_ clusters, atomic-resolution high-angle-annular-dark-field scanning transmission electron microscopy (HAADF-STEM) measurement was performed. The isolated bright dots in Fig. 2d can be assigned to NdCo_3_ clusters. Elemental mapping analysis of the STEM images revealed that the Nd, Co and P atoms are uniformly distributed throughout the nanosheets (Fig. 2e), demonstrating good dispersion of NdCo_3_ clusters on the PCN support.

**Figure 3.**
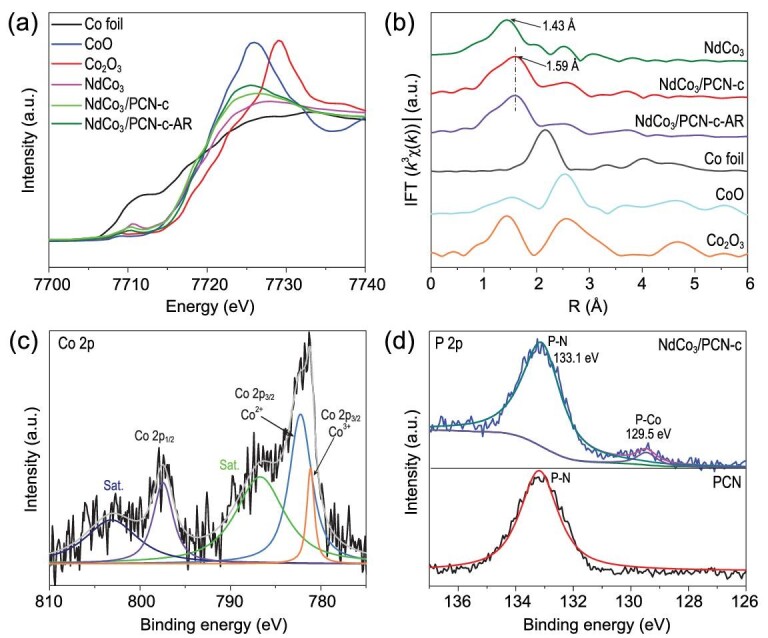
Characterization of NdCo_3_/PCN-c. (a) The Co K-edge XANES spectra and (b) corresponding Co k^3^-weighted FT spectra for Co foil, Co_2_O_3_, CoO, NdCo_3_, NdCo_3_/PCN-c and NdCo_3_/PCN-c-AR. (c) Co 2p XPS spectra of NdCo_3_/PCN-c. (d) P 2p XPS spectra of NdCo_3_/PCN-c and PCN.

Extended X-ray absorption fine structures (EXAFS) of NdCo_3_ and NdCo_3_/PCN-c were performed to probe the first coordination sphere of Co^3+^/Co^2+^ metal centers. As displayed in Fig. 3a, the Co K-edge X-ray absorption near edge spectroscopy (XANES) of the NdCo_3_ cluster gives a rising edge between that of CoO and Co_2_O_3_, indicating that Co centers in NdCo_3_ have mixed oxidation states of +2 and +3, which is consistent with the crystal structure analysis. The XANES spectrum of Co centers in NdCo_3_/PCN is very similar to that of the isolated NdCo_3_ cluster, suggesting that the Co oxidation states in NdCo_3_ remain the same during the assembly on PCN. The pre-edge of the NdCo_3_ and NdCo_3_/PCN-c showed that Co ions are maintaining an octahedral coordination [36]. As shown in Fig. 3b, the Fourier transform (FT) peak of the extended X-ray absorption fine structure (EXAFS) at 1.43 Å contains both Co-O and Co-N coordination. An emerging peak at 1.59 Å after anchoring the cluster on PCN could be ascribed to Co-P coordination with P from PCN. The EXAFS also confirms that no Co nanoparticles were formed in the reaction. A peak at a high R value (ca. 2.60 Å) corresponds to the distance of Co…Co path, which is also present in the as-prepared cluster. As shown in Supplementary Fig. 6, the experimental and fitting FT-EXAFS curve of Nd^3+^ (Nd L_III_ Edge) in NdCo_3_/PCN-c can be perfectly matched, which indicates that Nd^3+^ in NdCo_3_/PCN-c and the sample after reaction have the same coordination environment. The X-ray photoelectron spectroscopy (XPS) of NdCo_3_/PCN-c shows characteristic peaks of Nd 3d and Co 3d (Supplementary Fig. 7). Co 2p XPS spectra show different oxidation states of Co ions in the NdCo_3_ cluster on PCN nanosheets (Fig. 3c). The P 2p XPS spectra for the NdCo_3_/PCN-c sample displayed two peaks at 129.5 and 133.1 eV, which can be attributed to P with and without Co-P connections, respectively (Fig. 3d), and no peak related to Co-P showed up in the PCN spectra [36–38]. This XPS result suggests that P atoms coordinated with Co ion in the NdCo_3_ cluster. According to the EXAFS and XPS results, the NdCo_3_ cluster was anchored on the PCN through Co-P bonds and remained intact during the assembling process. The linker model of NdCo_3_/PCN-c was shown in Fig. 2a.

**Figure 4.**
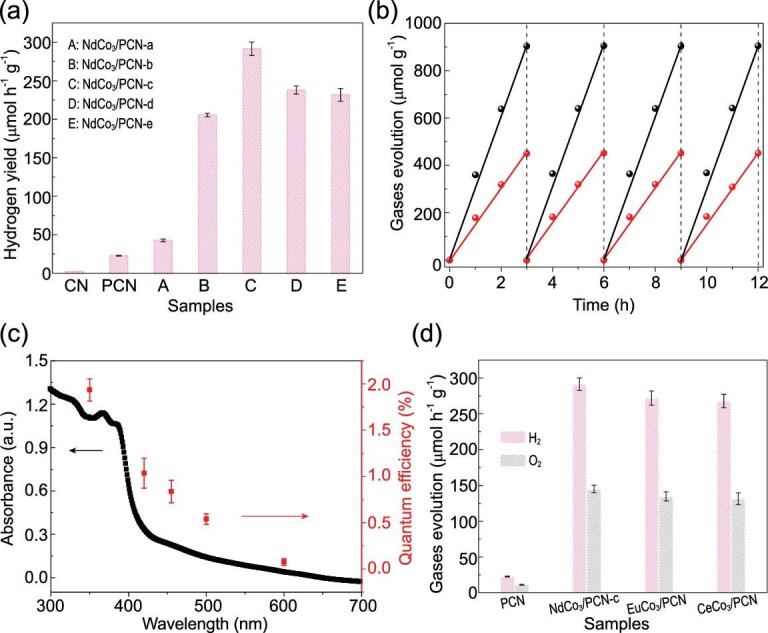
Photocatalytic performance of NdCo_3_/PCN-c. (a) H_2_ yield rates of CN, PCN, NdCo_3_/PCN-a, NdCo_3_/PCN-b, NdCo_3_/PCN-c, NdCo_3_/PCN-d and NdCo_3_/PCN-e. (b) Time course of H_2_ and O_2_ evolution of NdCo_3_/PCN-c for 12 hours. (c) The UV-vis absorption spectrum (black) and wavelength-dependent quantum efficiency (red dots) of water splitting (irradiated by a 300 W Xe lamp using a band-pass filter). (d) Gases evolutions of NdCo_3_/PCN-c, EuCo_3_/PCN and CeCo_3_/PCN.

The photocatalytic overall water-splitting performances of NdCo_3_/PCN-c catalysts were evaluated in pure water without any sacrificial reagents under simulated solar illumination (see details in Supplementary Data). As shown in Fig. 4a, NdCo_3_/PCN-c with different cluster loadings on PCN display different photocatalytic activities under light (λ > 300 nm) irradiation. With increased loading from 0.31 to 1.05 wt%, the photocatalytic activity of NdCo_3_/PCN-c improved because of the increased number of active sites for OER. The NdCo_3_/PCN-c with the loading of 1.05 wt% NdCo_3_ shows the highest photocatalytic H_2_ production rate of 297.7 μmol h^−1^ g^−1^ and O_2_ production rate of 148.9 μmol h^−1^ g^−1^, which is approximately 7.2 times that of PCN without loading clusters. Further increasing the loading of NdCo_3_ to 1.55 and 2.03 wt% leads to slightly reduced H_2_ and O_2_ production rates, possibly due to competitive transfer of holes to adjacent clusters, which decreases the chance of transferring four electrons to the same OEC to complete the whole OER process. As shown in Fig. 4b, the time courses of simultaneous evolution of H_2_ and O_2_ gases of NdCo_3_/PCN-c display a constant H_2_/O_2_ stoichiometric ratio of 2 : 1, suggesting the occurrence of overall water splitting. The NdCo_3_/PCN-c catalyst was recovered and reused four times without significant decrease in photocatalytic activity. A time course of H_2_ and O_2_ production of NdCo_3_/PCN-c under visible light irradiation (λ > 420 nm) was also studied. As displayed in Supplementary Figs 9 and 10, NdCo_3_/PCN-c shows about 210.4 μmol g^−1^ of H_2_ production rate and 105.7 μmol g^−1^ of O_2_ production rate in 12 hours under visible light irradiation. The quantum efficiency closely followed that of the ultraviolet-visible (UV-vis) absorbance trend, revealing that the reaction was driven by light absorption by the catalyst (Fig. 4c). Specifically, the NdCo_3_/PCN-c achieves a photocatalytic quantum efficiency of 2.0% at 350 nm and retains a quantum efficiency of 1.2% at the visible-light wavelength of 420 nm. To study the catalytic activity of NdCo_3_ itself, the cyclic voltammetry (CV) and linear sweep voltammetry (LSV) measurements were performed in the cell equipped with three electrodes, working electrode, counter electrode (Pt plate) and reference electrode (Ag/AgCl) in 0.5 M NaAc/HAc buffer solution (pH = 6). As shown in Supplementary Fig. 11, the NdCo_3_ cluster has obvious water oxidation catalytic activity. The overpotential for NdCo_3_ is 325 mV to reach 1 mA cm^−2^. The photocatalytic OER activity of the NdCo_3_ cluster itself was also studied in 20 mL 0.5 M NaAc/HAc (pH = 8) buffer solution using 1 mM [Ru(bpy)_3_]Cl_2_ as photosensitizer and 5 mM Na_2_S_2_O_8_ as sacrificial reagent. Under λ ≥ 420 nm light irradiation, the NdCo_3_ cluster shows a photocatalytic O_2_ production rate of 9.5 μmol h^−1^ g^−1^, which is close to the 11.5 μmol h^−1^ g^−1^ O_2_ evolutions of NdCo_3_/PCN-c (Supplementary Fig. 12). The close values of the photocatalytic O_2_ production rates of NdCo_3_ and NdCo_3_/PCN-c suggest that the OER rate is still the rate-determining step for the overall water splitting of NdCo_3_/PCN-c.

The TEM image and HAADF-STEM image of NdCo_3_/PCN-c after photocatalysis show that the morphology of NdCo_3_/PCN remained unchanged after the photocatalytic reaction (Supplementary Fig. 13). ICP-MS studies revealed that less than 0.3% of clusters leached into the solution after a reaction of 12 h, indicating the stability of NdCo_3_/PCN-c. The stability of clusters is not only due to the chelating effect of the bis-tris-propane ligand but also due to the Ln^3+^ ions that stabilize the 3d-4f cubane structure [39,40]. To verify the role of the lanthanide on the photocatalytic activities, the isostructural EuCo_3_/PCN and CeCo_3_/PCN clusters were also studied. As shown in Fig. 4d, under light (λ > 300 nm) irradiation, EuCo_3_/PCN and CeCo_3_/PCN show the photocatalytic H_2_ production rate of 279.1 and 274.5 μmol h^−1^ g^−1^ respectively, which are close to that of NdCo_3_/PCN-c. The time courses of H_2_ and O_2_ evolutions of EuCo_3_/PCN and CeCo_3_/PCN in 12 hours under light (λ > 300 nm) irradiation are displayed in Supplementary Figs 14 and 15. To exclude the contribution of other species for the catalytic activity in this system, the control experiments—by combining CoO, Co_3_O_4_ and Co(Ac)_2_, and Nd(NO_3_)_3_ with PCN as the photocatalysts respectively for overall water splitting based on the same method as that of NdCo_3_—were performed. As shown in Supplementary Fig. 16, CoO/PCN, Co_3_O_4_/PCN and Nd(NO_3_)_3_/PCN showed very low catalytic activity. Although the Co(Ac)_2_/PCN can give rise to a significant capability of water splitting (H_2_ production rate of 126.4 μmol h^−1^ g^−1^), compared with the activity of [Co(Ac)_2 _+ Nd(NO_3_)_3_]/PCN under the same conditions, the NdCo_3_/PCN-c shows much higher performance with 297.7 μmol h^−1^ g^−1^. These control experiments suggested that the NdCo_3_ itself boosts the activity in the system.

Electrochemical impedance spectroscopy (EIS) Nyquist plots and the transient photocurrent were measured to characterize the electron-hole transfer efficiency. NdCo_3_/PCN-c has a much smaller semicircle diameter and lower interfacial charge-transfer resistance than that of PCN, demonstrating the enhanced interfacial charge transfer of NdCo_3_/PCN-c (Supplementary Fig. 17). Consistently, NdCo_3_/PCN-c has better photocurrent responses under irradiation than that of PCN (Supplementary Fig. 18). Photoluminescence (PL) and the time-resolved fluorescence spectra of PCN and NdCo_3_/PCN-c were performed (Supplementary Fig. 19a). They were monitored at 430 nm under irradiation by a 368 nm laser at room temperature. Time-resolved fluorescence spectra revealed average lifetimes of approximately 2.17 and 1.91 ns for NdCo_3_/PCN-c and PCN, respectively (Supplementary Fig. 19b).

The photocatalytic H_2_ or O_2_ production reaction in the presence of a hole acceptor or electron acceptor could be performed to reveal more details about the two processes. The photocatalytic H_2_ evolution of NdCo_3_/PCN-c was enhanced in the presence of CH_3_OH as a hole acceptor, as compared to that without sacrificial agent (Supplementary Fig. 20), indicating that the intrinsic catalytic activity of the HER side is higher than that exhibited in overall water splitting. The rate-determining step is thus likely on the OER side. However, the photocatalytic O_2_ evolution of NdCo_3_/PCN-c in the presence of AgNO_3_ as an electron acceptor was slower than that without sacrificial agent (Supplementary Fig. 21), which suggests that the hole injection into NdCo_3_ is not the rate-determining step in the OER [41]. We thus conclude that the OER rate is still limited by catalysis.

**Figure 5.**
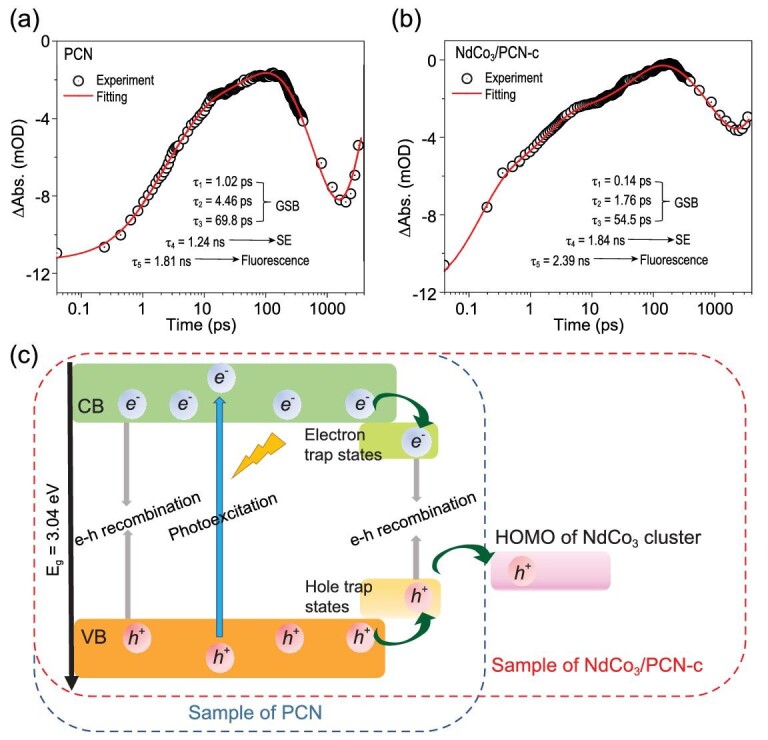
Spectroscopic evidence for effective charge separation process and DFT calculations of NdCo_3_/PCN-c. Representative ultrafast TA kinetics probed at 520 nm (pumped at 360 nm) for (a) PCN and (b) NdCo_3_/PCN-c. The TA signal (i.e. the absorbance changes, or ΔAbs. in short) is given in mOD where OD stands for optical density. (c) Schematic illustration of the mechanism involved.

Femtosecond time-resolved transient absorption (fs-TA) spectroscopy was used to detect the ultrafast excited state dynamics of the system (Supplementary Fig. 22) [42,43]. The dynamics in the femtosecond-picosecond (fs-ps) time scale can be fitted to a five-component exponential model as shown by the time trace at 520 nm (excited at 360 nm) (Fig. 5a and b). In the initial 100 ps, negative signals due to ground state bleach (GSB) are prominent, which reflect the behavior of holes on the valence band (VB). Evolution of the initial GSB signal can be described by three time constants: τ_1_ = 1.02 ps, τ_2_ = 4.46 ps, τ_3_ = 69.8 ps for PCN, and τ_1_ = 0.14 ps, τ_2_ = 1.76 ps, τ_3_ = 54.5 ps for NdCo_3_/PCN-c. The τ_1_ and τ_2_ correspond to initial vibrational cooling of energetic holes, and τ_3_ may be attributed to the hole transfer process to the surface trap site. Compared to the PCN sample in the early 100 ps, NdCo_3_/PCN-c has shorter relaxation times of all the three components. The accelerated hole transfer rate may be related to fast hole transfer to the NdCo_3_ cluster in NdCo_3_/PCN-c. After a few hundred picoseconds, electrons and holes complete the transfer to trap states. The TA signals of both PCN and NdCo_3_/PCN-c samples showed a significant signal growth on the timescale of hundreds of ps to ns (τ_4_). This growth may be due to stimulated emission (SE) from trap states, which are supported by fluorescence lifetimes on an ns timescale. The NdCo_3_/PCN-c sample shows a longer τ_4_ time than PCN (τ_4_ = 1.84 ns for NdCo_3_/PCN-c vs. τ_4_ = 1.24 ns for PCN) because hole transfer to the cluster competes against populating the surface trap sites and thus delays the emissive electron-hole recombination. The fifth time constant (τ_5_ = 2.39 ns for NdCo_3_/PCN-c vs. τ_5_ = 1.81 ns for PCN) represents the fluorescence process, which is consistent with the result from time-resolved fluorescence (Supplementary Fig. 19b). The τ_5_’s determined by TA spectra are not very accurate due to the limited number of points on the long waiting time. Based on these analyses, we can understand the reason why NdCo_3_/PCN-c improves higher photocatalytic performance (Fig. 5c). The cluster not only acts as a reaction center for water oxidation but also suppresses electron-hole recombination due to fast hole injection into the clusters. This efficient hole transfer leads to an increase in hole utilization and finally improves the overall efficiency.

To further investigate the catalytic OER, spin polarized DFT + U calculations were carried out using the VASP software [44]. NdCo_3_ clusters can easily lose two Ac^−^ ligands from the Nd^3+^ and Co^3+^ ions in aqueous solution, resulting in two coordination unsaturated sites (CUS). A series of geometrical optimizations reveal that the CUS of one Co (III) ion prefers to coordinate with PCN by the anchoring site of P atom (d_P-Co_ = 2.436 Å) with an adsorption energy of −1.01 eV, while another Co^3+^(CUS) can serve as the catalytic center. One water molecule adsorbs on the CUS of Co^3+^ ions with a d_Co-O_ of 2.191 Å and adsorption energy (E_ads_) of −0.74 eV. Both the charge density difference and electron localization function (ELF) value (around 0.5) suggest that the 3d orbital of Co^3+^ (CUS) ions effectively overlaps with the 2p orbital of O_w_, resulting in the formation of one weak coordination bond. Partial density of states curves of spin up and spin down indicate that spin carriers (e.g. Nd and Co ions, P and N of C_3_N_4_) present apparent spin polarization at the vicinity of Fermi level (Supplementary Fig. 25). Moreover, possible reaction intermediate species of ^*^OH, ^*^O, ^*^OOH exhibit strong adsorption on this reaction center (E_ads_ = −3.14 eV for ^*^OH, −3.94 eV for ^*^O and −1.22 eV for ^*^OOH). While H_2_O and ^*^OH only locate on the CUS of a Co^3+^ ion, ^*^O and ^*^OOH attach to both the Co^3+^ ion and Nd^3+^ ion. The highly charged Nd^3+^ effectively stabilizes these electron-rich intermediates to facilitate water oxidation.

Oxidation states of metal ions are poorly described by Bader charge calculation [45]. As a result, magnetic moments were evaluated to assist oxidation state identification, considering their similar coordination field from oxygen atoms [46]. The spin states of each intermediate were investigated by using symmetry-broken calculations (Supplementary Table 5). Initially, both Co (P) and Co (CUS) ions feature low spin state Co^III^ ions, while the middle divalent Co(M) ion features high spin state Co^II^ ion. After two hole injections to the cluster, the water molecule attached to the Co^III^(CUS) ion is deprotonated and converted to ^*^OH. The injected two holes bring the Co^III^(P), Co^II^(M) and Co^III^(CUS) ions to the oxidation states of +4, +3 and +3, respectively. Then, another hole injection induced the deprotonation of ^*^OH, yielding bridging ^*^O in Co^IV^(CUS)-oxo-Nd^III^ (spin magnetization of 2.875 μ_B_ on Co). The Co^IV^(CUS)-oxo is electron deficient, which is an ideal target for a nucleophilic attack by a second water in a concerted process of forming one O−O bond and one O-Nd^III^ coordination while losing one proton upon injection of another hole. A bridging ^*^OOH species and high oxidation state Co^V^(CUS)-hydroperoxyl-Nd^III^ (spin magnetization of −2.648 μ_B_ on Co) is formed. Finally, the liberation of O_2_ from the cluster with concomitant deprotonation and reduction of the cluster to regenerate its initial oxidation state happens at low activation energy. Generally speaking, the Ln^3+^ ion stabilizes negatively charged intermediates, and the other two Co ions that are not directly attached to water molecules serve as hole reservoirs to store oxidation equivalents and thus avoid the building up of too high an oxidation potential on one Co ion. All four metal ions in the cluster synergistically catalyze the water oxidation (Supplementary Fig. 28).

## CONCLUSION

In summary, we demonstrated a bio-inspired lanthanide-transition metal cluster as an oxygen-evolving center anchored on PCN for efficient photocatalytic overall water splitting. The obtained LnCo_3_ clusters not only display high stability but also show excellent oxygen-evolving activity. The combination of LnCo_3_ clusters and PCN achieves efficient separation of electrons and holes and enables rapid production of H_2_ and O_2_. Mechanistic investigation shows synergistic effects of lanthanide ion and variable-valence Co ions in the oxygen-evolving reaction. This work not only prepares a synthetic model of a bio-inspired oxygen-evolving center but also develops an avenue to designing efficient catalysts for overall water splitting by coupling bio-inspired clusters and photoactive supports.

## METHODS

### Synthesis of [NdCo_3_(btp-3H)_2_(Ac)_2_(NO_3_)_2_]·(NO_3_)·2H_2_O

A mixture of Nd(NO_3_)_3_**·**6H_2_O (0.438 g, 1 mmol), Co(Ac)_2_**·**4H_2_O (0.125 g, 0.5 mmol) and btp (0.141 g, 0.5 mmol) was dissolved in methanol (10.0 mL), followed by the addition of trimethylamine (150 μL). The mixture was heated to reflux for 40 minutes and then filtered after cooling. Lamella-shaped brown crystals of [NdCo_3_(btp-3H)_2_(Ac)_2_(NO_3_)_2_]·(NO_3_)·2H_2_O (**1**) were obtained in 35% yield (based on Nd(NO_3_)_3_**·**6H_2_O) after the filtrate was kept at room temperature for 1 week. For C_26_H_56_N_7_Co_3_NdO_27_ (FW = 1219.8): C, 25.60; H, 4.63; N, 8.04. Found: C, 25.43; H, 4.85; N, 8.08.

### Synthesis of P-doped C_3_N_4_ photocatalysts

A mixture of 0.5 g of the prepared C_3_N_4_ and 0.25 g NaH_2_PO_2_ was ground with motar. Then, the mixture was heated to 350^o^C in 2^o^C/min in a muffle furnace and then heated for 2 h in a N_2_ atmosphere. The resultant precipitate was ultrasonicated and washed with water and ethanol twice, collected by filtration and dried at 70^o^C overnight.

### Synthesis of NdCo_3_/PCN photocatalysts

Forty-five milligrams of PCN was dispersed in methanol solution (3 mg/mL) with sonication, and then transferred to a flask with stirring, then 1 mg, 2 mg, 3 mg, 5 mg and 7 mg NdCo_3_ clusters in 1 mL methanol were dropped into the suspension and refluxed for 12 h, respectively. The resultant precipitate was collected by filtration and dried at 70^o^C overnight.

### Photocatalytic reactions

The photocatalytic experiments were performed via a photocatalytic evaluation system (CEL-SPH2N, CEAULight, China) in a 300 mL Pyrex flask. A 300 W Xenon arc lamp with a wavelength range of 300–800 nm was used as the light source. The focused intensity on the flask was ∼200 mW · cm^−2^. In a typical photocatalytic experiment, 40 mg of photocatalyst was suspended in aqueous solution. Before irradiation, the system was vacuumed for 10 min via the vacuum pump to completely remove the dissolved oxygen. The evolved gases contents were analyzed by gas chromatography (GC7920, CEAULight, China). The apparent quantum efficiency was measured under identical photocatalytic reactions. Single wavelength 365 nm, 420 nm, 450 nm, 500 nm and 600 nm filters were employed as the light sources to trigger the photocatalytic reactions, respectively.

### Photochemical studies

Cyclic voltammograms (CV), EIS data, photocurrent and the Mott–Schottky spots were recorded using electrochemical workstation (CHI 760E, Shanghai Chenhua). The Indium tin oxide glasses with samples were served as the working electrodes. EIS measurements were recorded over a frequency range of 100 kHz–200 kHz with ac amplitude of 20 mV at 0 V. Water was used as the supporting electrolyte. The Mott-Schottky plots were also measured over an alternating current frequency of 1000 Hz, 1200 Hz and 1500 Hz. These three electrodes were immersed in the 0.2 M Na_2_SO_4_ aqueous solution (pH = 6.6).

All other experimental details, as well as TA spectroscopy characterizations and the DFT calculations, are provided in the Supplemental Experimental Procedures.

## Supplementary Material

nwaa234_Supplemental_Files
