# Supplementary material for: Integration of bio-inspired lanthanide-transition metal cluster and P-doped carbon nitride for efficient photocatalytic overall water splitting
Source: Natl Sci Rev. 2020 Sep 14;8(9):nwaa234. doi: 10.1093/nsr/nwaa234 (PMC8433082; doi:10.1093/nsr/nwaa234)
Supplement: nwaa234_Supplemental_Files [file nwaa234_supplemental_files.zip › Supporting_Information.docx]

**Supporting Information**

**Integration of Bio-Inspired Lanthanide-Transition** **Metal Cluster and P-doped Carbon Nitride for Efficient Photocatalytic Overall Water Splitting**

Rong Chen, ^1,†^ Gui-Lin Zhuang,^2,†^ Zhi-Ye Wang,^1^ Yi-Jing Gao,^2^ Zhe Li,^1^ Cheng Wang,^1^ Yang Zhou,^1^ Ming-Hao Du,^1^ Suyuan Zeng,^3^ La-Sheng Long,^1^ Xiang-Jian Kong,^1,^* and Lan-Sun Zheng^1^

*1 Collaborative Innovation Center of Chemistry for Energy Materials, State Key Laboratory of Physical Chemistry of Solid Surface and Department of Chemistry, College of Chemistry and Chemical Engineering, Xiamen University, Xiamen 361005, China*

*2 College of Chemical Engineering, Zhejiang University of Technology, 310032 Hangzhou, China.*

*3 College of chemistry and chemical engineering, Liaocheng University, Liaocheng, 252059, China*

*^†^ These authors contributed equally*

** Correspondence:* [*xjkong@xmu.edu.cn*](mailto:xjkong@xmu.edu.cn)

**Table of Contents**

**Experimental Section S3**

**Characterization S4**

**Single Crystal X-ray Diffraction Determination of single crystals of 1-3 S5**

**Supplementary Table 1:** **Single Crystal X-ray Structure Refinement S6**

**X-ray absorption spectroscopy (XAS) measurements S6**

**Supplementary Table 2: Summary of EXAFS fitting parameters S6**

**Photocatalytic overall water splitting and Photochemical studies S7**

**TA spectroscopy characterizations S8**

**Supplementary Table 3: The constants of the TA spetra S9**

**Supplementary Figure 1: The [Co^III^(btp-3H)] unit of LnCo_3_ cluster S10**

**Supplementary Figure 2: IR spectra of NdCo_3_, EuCo_3_ and CeCo_3_ cluster S10**

**Supplementary Figure 3: ESI-MS spectra of NdCo_3_ cluster S11**

**Supplementary Figure 4: ESI-MS spectra of EuCo_3_ and CeCo_3_ clusters S11**

**Supplementary Figure 5: IR spectra of PCN and NdCo_3_/PCN-c S12**

**Supplementary Figure 6: Comparison of Nd^3+^ FT-EXAFS curves S12**

**Supplementary Figure 7: Full XPS spectra of LnCo_3_/PCN-c S13**

**Supplementary Figure 8: C 1s and N 1s XPS of PCN and NdCo_3_/PCN-c S13**

**Supplementary Figure 9: A time course of H_2_ and O_2_ production S14**

**Supplementary Figure 10: The gases evolutions of NdCo_3_/PCN-c S14**

**Supplementary Figure 11: CV and LSV of NdCo_3_ S15**

**Supplementary Figure 12: Photocatalytic activity of NdCo_3_ S15**

**Supplementary Figure 13: TEM image and HAADF-STEM image S16**

**Supplementary Figure 14: H_2_ and O_2_ production of EuCo_3_/PCN S16**

**Supplementary Figure 15: H_2_ and O_2_ production of CeCo_3_/PCN S16**

**Supplementary Figure 16: The comparison hydrogen yield S17**

**Supplementary Figure 17: EIS of PCN and NdCo3/PCN-c in water S17**

**Supplementary Figure 18: Transient photocurrent response S17**

**Supplementary Figure 19: PL and Time-resolved fluorescence spectra S18**

**Supplementary Figure 20: The photocatalytic H_2_ production S18**

**Supplementary Figure 21: The photocatalytic O_2_ production S18**

**Supplementary Figure 22: Representative TA spectra S19**

**Supplementary Figure 23: XPS spectra S19**

**Computational Details S20**

**Supplementary Figure 24: Experiment plots of χ_m_T vs T S22**

**Supplementary Figure 25: Adsorption structure S22**

**Supplementary Figure 26: Partial density of states S22**

**Supplementary Figure 27: Relation curve of electronic occupations S23**

**Supplementary Figure 28: Proposed mechanism S23**

**Supplementary Table 4: The fitting of Time-resolved fluorescence spectra S24**

**Supplementary Table 5: Spin projection magnetization of selective atoms S24**

**Supplementary Table 6: Summary of the mass ratios and H_2_ production S24**

**Supplementary Table 7: Summary of the mass ratios and H_2_ production S25**

**Supplementary Table 8: Selected Bond distances (Å) of compound NdCo_3_ S25**

**Supplementary Table 9: Selected Bond distances (Å) of compound EuCo_3_ S26**

**Supplementary Table 10: Selected Bond distances (Å) of compound CeCo_3_ S26**

**Supplementary Table 11. The calculated states of the metal ions S27**

**Supplementary Table 12. The calculated states of atoms in the ligand S28**

**References S29**

**Experimental Section**

**1.1 Chemicals:** BIS-TRIS-propane (btp), Cobalt acetate tetrahydrate **(**Co(Ac)_2_**·**4H_2_O), Neodymium(III) nitrate hexahydrate (Nd(NO_3_)_3_**·**6H_2_O), Europium(III) nitrate hexahydrate (Eu(NO_3_)_3_**·**6H_2_O), Cerium(III) nitrate hexahydrate (Ce(NO_3_)_3_**·**6H_2_O), Triethylamine (NEt_3_), Methanol (CH_3_OH) are analytical grade, and used as received without further purification.

**1.2 Synthesis of [NdCo_3_(btp-3H)_2_(Ac)_2_(NO_3_)_2_]·(NO_3_)·2H_2_O (1)** A mixture of Nd(NO_3_)_3_**·**6H_2_O (0.438 g, 1 mmol), Co(Ac)_2_**·**4H_2_O (0.125 g, 0.5 mmol), and bis-tris-propane (btp, 0.141 g, 0.5 mmol) were dissolved in methanol (10.0 mL); followed by the addition of trimethylamine in the above mixture until to the point of incipient but permanent precipitation. The mixture was heated to reflux for 40 minutes and then filtered after cooling. Lamella-shaped brown crystals of **1** were obtained in 35% yield (based on Nd(NO_3_)_3_**·**6H_2_O) after the filtrate was kept at room temperature for 1 week. For C_26_H_56_N_7_Co_3_NdO_27_ (FW = 1219.8): C, 25.60; H, 4.63; N, 8.04. Found: C, 25.43; H, 4.85; N, 8.08.

**1.3 Synthesis of [EuCo_3_(btp-3H)_2_(Ac)_2_(NO_3_)_2_]·(NO_3_)·4H_2_O (2)** Compound **2** was synthesized by the same method except the substitution of Nd(NO_3_)_3_**·**6H_2_O to Eu(NO_3_)_3_**·**6H_2_O. For C_26_H_60_N_7_Co_3_EuO_29_ (FW = 1263.55): C, 24.70; H, 4.78; N, 7.76. Found: C, 24.52; H, 4.68; N, 7.62.

**1.4 Synthesis of [CeCo_3_(btp-3H)_2_(Ac)_2_(NO_3_)_2_]·(NO_3_)·2H_2_O (3)** Compound **3** was synthesized by the same method except the substitution of Nd(NO_3_)_2_**·**6H_2_O to Ce(NO_3_)_2_**·**6H_2_O. For C_26_H_56_N_7_CeCo_3_O_27_ (FW = 1215.67): C, 25.69; H, 4.64; N, 8.06. Found: C, 25.34; H, 4.58; N, 7.89.

**1.5 Synthesis of** **C_3_N_4_ photocatalyst** 35 g urea was put into a covered crucible and then heated at 550^o^C for 4 h in a muffle furnace at a ramp rate of 5^o^C/min. The result precipitate was about 2.2 g, and was ultrasonicated with deionized water for 1 h, collected by centrifugation and dried at 70^o^C overnight.

**1.6 Synthesis of P-doped C_3_N_4_ photocatalyst (PCN)** A mixture of 0.5 g the prepared C_3_N_4_ and 0.25 g NaH_2_PO_2_ grind with motar. Then, the mixture was heated to 350 ^o^C in 2^o^C /min in muffle furnace and then heated for 2 h in a N_2_ atmosphere. The resultant precipitate was ultrasonicated and washed with water and ethanol for two times, collected by filtration and dried at 70^o^C overnight.

**1.7 Synthesis of NdCo_3_/PCN photocatalysts** 45 mg PCN was dispersed in methanol solution (3 mg/mL) with sonication, and then transferred to flask with stirring, then 1 mg, 2 mg, 3 mg, 5 mg and 7 mg NdCo_3_ cluster in 1 mL methanol were dropped into the suspension and refluxed for 12 h, respectively. The resultant precipitate was collected by filtration and dried at 70^o^C overnight.

**1.8 Synthesis of EuCo_3_/PCN, CeCo_3_/PCN photocatalysts** 45mg PCN was dispersed in methanol solution (3 mg/mL) with sonication, and then transferred to flask with stirring, then 3 mg EuCo_3_ or CeCo_3_ clusters in 1 mL methanol were dropped into the suspension and refluxed for 12 h, respectively. The resultant precipitate was collected by filtration and dried at 70^o^C overnight.

**2. Characterization**

The transmission electron microscopy (TEM) and high-resolution TEM (HRTEM) images were recorded on Talos F200X (Thermo Fischer). UV-visible diffused reflectance spectra were recorded using a Cary 5000 UV-Vis spectrometer (Viarian, USA) with BaSO_4_ as a reflectance standard. The photoluminescence spectra were performed on a Molecular Fluorescence Spectrometer (F7000). Time-resolved PL decay curves were obtained on a FLS980 fluorescence lifetime spectrophotometer (Edinburgh Instruments, UK) under the excitation of 368 nm and probed at 430 nm. XPS spectra were obtained on a ESCALAB 250Xi. The binding energy was calibrated by means of the C 1s peak energy of 284.6 eV. EPR spectra were obtained over Bruker EMX-10/12 electron paramagnetic resonance spectrometer at room temperature. Inductively coupled plasma-mass spectrometry (ICP-MS) data were obtained with an Agilent 7700x ICP-MS and analyzed using ICP-MS MassHunter version B01.03. Samples were diluted in a 2% HNO_3_ matrix and analyzed with a ^159^Tb internal standard against a 10-point standard curve over the range from 1 ppb to 500 ppb. Microanalyses of C, H and N were carried out with a CE instruments EA 1110 elemental analyzer. An infrared spectrum was recorded on a Nicolet AVATAR FT-IR360 spectrophotometer with pressed KBr pellets. An infrared spectrum was recorded on a Nicolet AVATAR FT-IR360 spectrophotometer with pressed KBr pellets. Mass spectrum was recorded on an Agilent Technologies ESI-TOF-MS.

1. **Single Crystal X-ray Diffraction Determination of single crystals of 1-3**

Data of the clusters **1-3** were collected on a Oxford Gemini S Ultra diffractometer using graphite monochromatized MoKα radiation (λ= 0.71073 Å) at 173 K. The structures were solved by direct methods (SHELXTL Version 6.12), and the non-hydrogen atoms were refined anisotropically by full-matrix least-squares method on F^2^. The hydrogen atoms of organic ligand were generated geometrically (C-H = 0.96 Å, N-H = 0.90 Å). Crystal data, as well as details of the data collection and refinement, for the complexes are summarized in **Supplementary Table 1**. CCDC numbers of 1954753-1954755 for **1**-**3** contain the supplementary crystallographic data for this paper.

**Supplementary Table 1.** Single Crystal X-ray Structure Refinement of **1-3**.

| Complex | **1** | **2** | **3** |
| --- | --- | --- | --- |
| Formula | C_26_H_56_N_7_Co_3_NdO_27_ | C_26_H_60_N_7_Co_3_EuO_29_ | C_26_H_56_N_7_Co_3_CeO_27_ |
| Mr | 1219.8 | 1263.55 | 1215.67 |
| T(K) | 173(2) | 173(2) | 173(2) |
| cryst syst | monoclinic | monoclinic | orthorhombic |
| space group | *P*2_1_*/n* | *P*2_1_*/n* | *Cmce* |
| A, Å | 10.0829(3) | 10.0691(3) | 27.895(2) |
| B, Å | 25.4908(8) | 25.5194(10) | 19.2713(13) |
| C, Å | 16.5392(6) | 16.5217(4) | 17.1443(11) |
| V, Å^3^ | 4250.8(2) | 4244.9(2) | 9216.4(11) |
| Z, | 3 | 3 | 1 |
| Dc, (g cm^-3^) | 1.827 | 2.847 | 0.044 |
| µ, (mm^-1^) | 57.556 | 8.771 | 1.277 |
| data/params | 6154/622 | 8510/610 | 3494/281 |
| θ (deg) | 6.936-131.312 | 5.876-55.466 | 7.594-121.016 |
| obsd reflns | 10358 | 17887 | 8886 |
| *R*_1_^[a]^[I>2σ (I )] | 0.0674 | 0.1173 | 0.1146 |
| *wR*_2_^[b]^ (all data) | 0.1550 | 0.2097 | 0.2667 |

[a] R_1_ = ∑ | |Fo| - |Fc| | / ∑ |Fo|. [b] wR_2_ = {∑ [w (Fo^2^ – Fc^2^)^2^] / ∑ [w(Fo^2^)^2^]}^1/2^.

1. **X-ray absorption spectroscopy (XAS) measurements**

XAS measurements were carried out at the BL14W1 beamline of the Shanghai Synchrotron Radiation Facility (SSRF) operated at 3.5 GeV under “top-up” mode with a constant current of 260 mA. Spectra were collected at the cobalt K-edge (7709 eV) in transmission mode. The X-ray beam was monochromatized by a Si (111) monochromater and detuned by 50% to reduce the contribution of higher-order harmonics below the level of noise. A metallic cobalt foil standard was used as a reference for energy calibration and was measured simultaneously with experimental samples. Three X-ray absorption spectra were collected at room temperature for each sample. Data was processed using the Athena and Artemis programs of the IFEFFIT package based on FEFF 6.3-4 prior to merging, spectra were calibrated against the reference spectra and aligned to the first peak in the smoothed first derivative of the absorption spectrum, the background noise was removed, and the spectra were processed to obtain a normalized unit edge step. Fitting result was shown in Fig. S6A. For fitting parameters, see **Supplementary Table 2**.

**Supplementary Table 2.** Summary of EXAFS fitting parameters for NdCo_3_/PCN-c (Nd L_III_ Edge).

| **NdCo_3_/PCN-c** | | Fitting Range | R: 1 – 3.2 Å |
| --- | --- | --- | --- |
| Independent Points | 10 | Variables | 6 |
| Reduced chi-square | 113 | R-factor | 0.018 |
| ΔE_0_(eV) | 9.83 | S_0_^2^ | 0.85 |
| R(Nd-O^1^) (**1**) | 2.43 ± 0.01 Å | σ^2^(Nd-O^1^) | 0.013 ± 0.001 |
| R(Nd-O^2^) (**1**) | 2.47 ± 0.01 Å | σ^2^ (Nd-O^2^) | 0.013 ± 0.001 |
| R(Nd-O^3^) (**1**) | 2.52 ± 0.01 Å | σ^2^ (Nd-O^3^) | 0.013 ± 0.001 |
| R(Nd-O^4^) (**1**) | 2.54± 0.01 Å | σ^2^ (Nd-O^4^) | 0.013 ± 0.001 |
| R(Nd-N) (**1**) | 2.95 ± 0.15 Å | σ^2^ (Nd-N) | 0.003 ± 0.002 |
| R(Nd-Co) (**1**) | 3.19 ± 0.16 Å | σ^2^(Nd-Co) | - 1. ± 0.002 |

1. **Photocatalytic overall water splitting**

The photocatalytic experiments were performed via a photocatalytic evaluation system (CEL-SPH2N, CEAULight, China) in a 300 mL Pyrex flask, and the openings of the flask were sealed with silicone rubber septum. A 300 W xenon arc lamp with a wavelength range of 300-800 nm was used as the light source. The focused intensity on the flask was about 200 mW·cm^−2^. In a typical photocatalytic experiment, 40 mg of the prepared photocatalyst was suspended in aqueous solution. Before irradiation, the system was vacuumed for 10 min via the vacuum pump to completely remove the dissolved oxygen and ensure the reactor was in a vacuum condition. The evolved gases contents were analyzed by gas chromatography (GC7920, CEAULight, China). The apparent quantum efficiency (QE) was measured under the identical photocatalytic reactions. Single wavelength 365 nm, 420 nm, 450 nm, 500 nm and 600 nm filters were employed as the light sources to trigger the photocatalytic reactions, respectively. The quantum efficiency (QE) was calculated according to the following equation:

$$\begin{matrix} \text{QE(\%)}=\frac{\text{Number of reacted electrons}}{\text{Number of incident photons}}\times100 \\ \text{ }=\frac{\text{Number of evolved H}_{2}\text{molecules}\times2}{\text{Number of incident photons}}\times100 \end{matrix}$$

1. **The photochemical studies**

Cyclic voltammograms (CV), Electrochemical Impedance Spectroscopy (EIS) data, photocurrent and the Mott–Schottky spots were recorded using Electrochemical workstation (CHI 760E, Shanghai Chenhua). The clean and dry Indium tin oxide (ITO) glasses served as the working electrodes. 3 mg catalysts were dispersed in the 0.65 mL ethanol, 0.25 mL isopropanol and 100 μL naphthol and then sonication for producing slurry. The conductive tape was used to adhere to part of ITO glasses to leave an area of 1×1 cm^2^ for depositing 40 μL slurry. And then, the working electrode with 40 μL slurry was dried at room temperature naturally. The electrochemical measurements were performed in the cell equipped with three electrodes, namely working electrode, counter electrode (Pt plate) and reference electrode (Ag/AgCl). Electrochemical impedance spectra (EIS) measurements were carried out in three-electrode system and recorded over a frequency range of 100 kHz-200 kHz with ac amplitude of 20 mV at 0 V. Water was used as the supporting electrolyte. Photocurrent was measured in the same three-electrode system. A 300W Xenon light was applied as the light source. Water was used as the electrolyte. The Mott-Schottky plots were also measured using the same three electrode system over an alternating current (AC) frequency of 1,000 Hz, 1,200 Hz and 1,500 Hz. These three electrodes were immersed in the 0.2 M Na_2_SO_4_ aqueous solution (pH = 6.6).

1. **TA spectroscopy characterizations**

The femtosecond transient absorption system is based on Coherent's regeneratively amplified titanium-doped gemstone femtosecond laser (center wavelength 800 nm, pulse width 35 fs, exit light intensity 5.8 mJ/pulse, repetition rate 1 kHz), optical parametric amplifier and home-built delay and detection systems with Andor spectrometers. All the measurements were performed under ambient conditions. The sample cell is a flow cell to keep the sample fresh during the experiment.

**Table S3.** The constants of the TA spectra of PCN and NdCo_3_/PCN-c.

|  | *A1* | *τ_1_(ps)* | *β_2_* | *τ_2_(ps)* | *β_3_* | *τ_3_(ns)* | *β_4_* | *τ_4_(ns)* | *β_5_* | *τ_5_(ns)* |
| --- | --- | --- | --- | --- | --- | --- | --- | --- | --- | --- |
| **PCN** | -0.00294 | 1.03 | -0.00489 | 4.45 | -0.00423 | 0.070 | 0.06108 | 1.23 | -0.0602 | 1.8 |
| **NdCo_3_/PCN-c** | -0.00472 | 0.14 | -0.00346 | 1.76 | -0.00339 | 0.054 | 0.039 | 1.83 | -0.03834 | 2.4 |

**
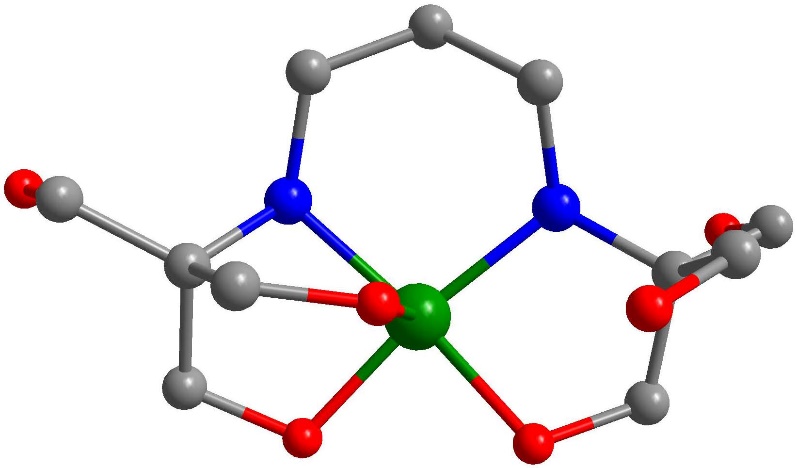
**

**Supplementary Figure 1 |** The [Co^III^(btp-3H)] unit of LnCo_3_ cluster.


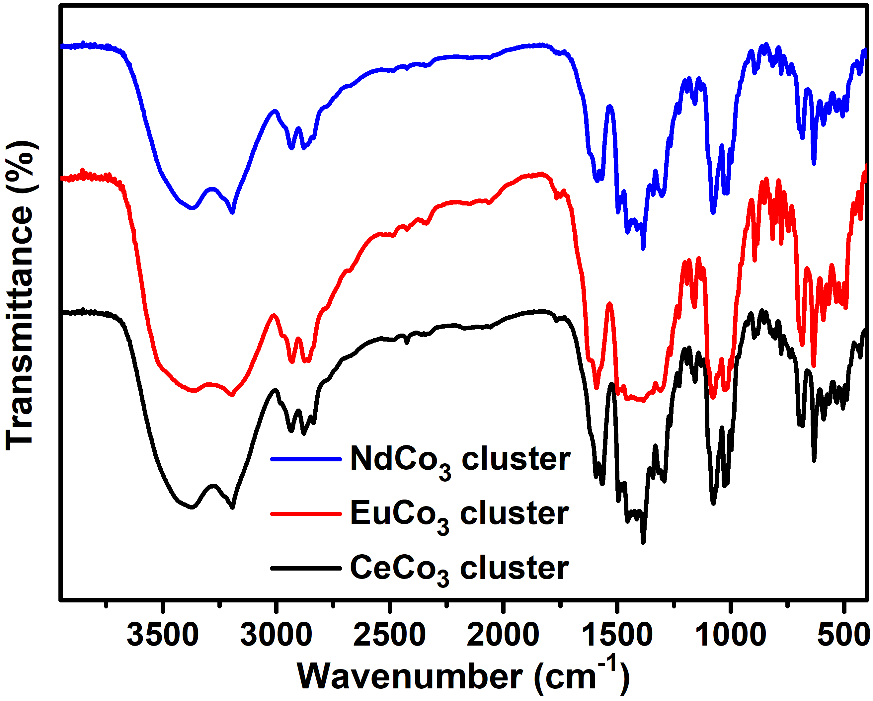


**Supplementary Figure 2 |** FT-IR spectra in 400-4000 cm^-1^ of NdCo_3_, EuCo_3_ and CeCo_3_ clusters.


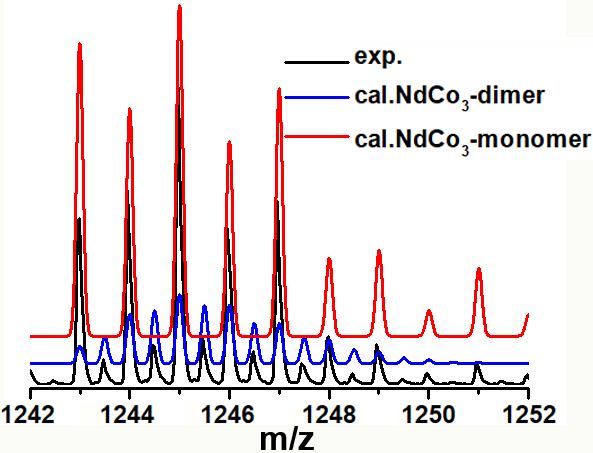


**Supplementary Figure 3 |** ESI-MS spectra of NdCo_3_ cluster in the range from 1242 to 1252 (m/z).


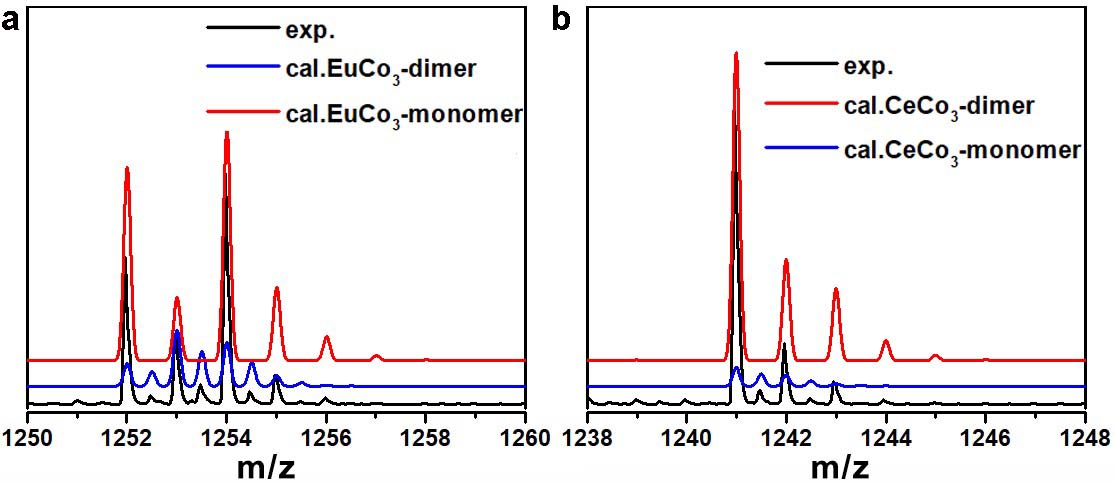


**Supplementary Figure 4 |** ESI-MS spectra of (a) EuCo_3_ in the range from 1250 to 1260 (m/z) and (b) CeCo_3_ in the range from 1238 to 1248 (m/z).


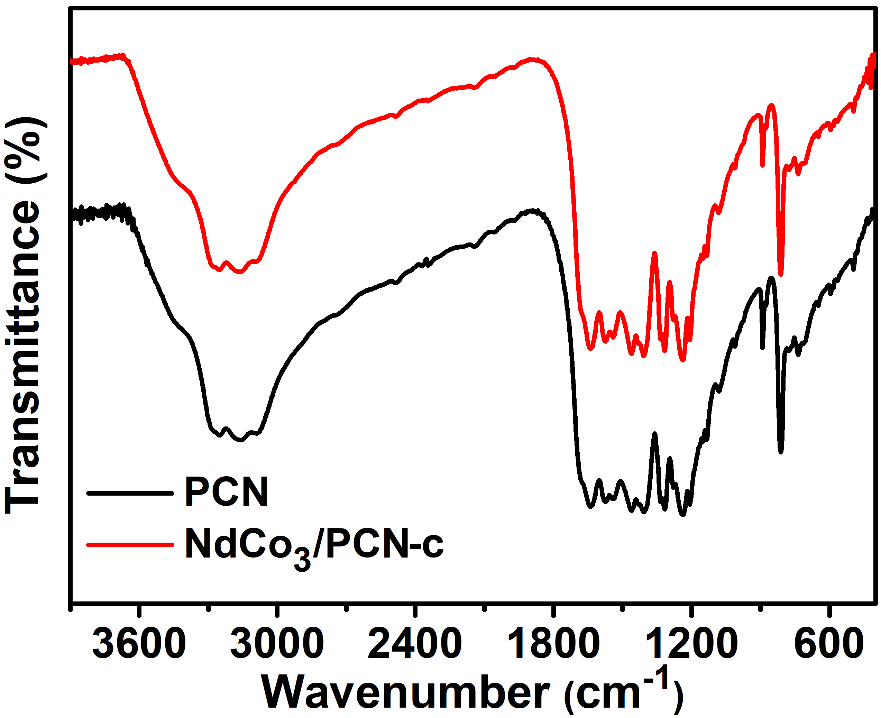


**Supplementary Figure 5 |** FT-IR spectra of PCN and NdCo_3_/PCN-c. The typical C-N heterocycle stretches in the 1200-1600 cm^-1^ region and the breathing mode of the heptazine units at 800 cm^-1^ is very similar. In addition, the broad peak at 3000-3500 cm^-1^ was ascribed to the stretching vibrations of N-H and O-H resulted from physically adsorbed water.


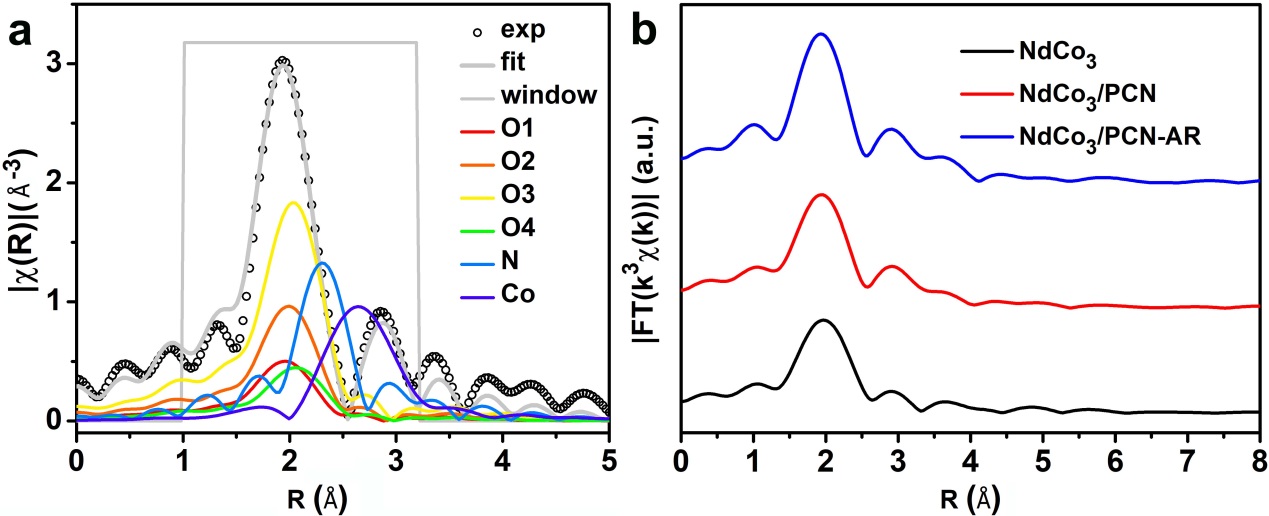


**Supplementary Figure 6 |** (a) Comparison of Nd^3+^ FT-EXAFS curves between the experimental data and the fit of NdCo_3_/PCN-c; (b) The corresponding Nd k^3^-weighted FT spectra of NdCo_3_, NdCo_3_/PCN-c and NdCo_3_/PCN-c-AR (after reaction).


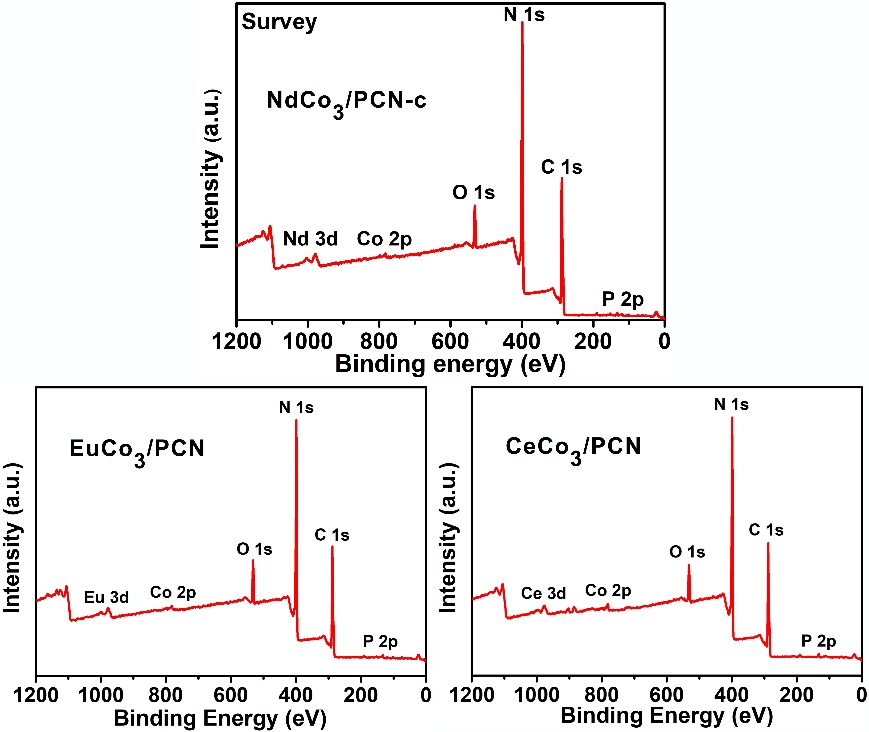


**Supplementary Figure 7 |** Full XPS spectra of NdCo_3_/PCN-c, EuCo3/PCN and CeCo_3_/PCN.


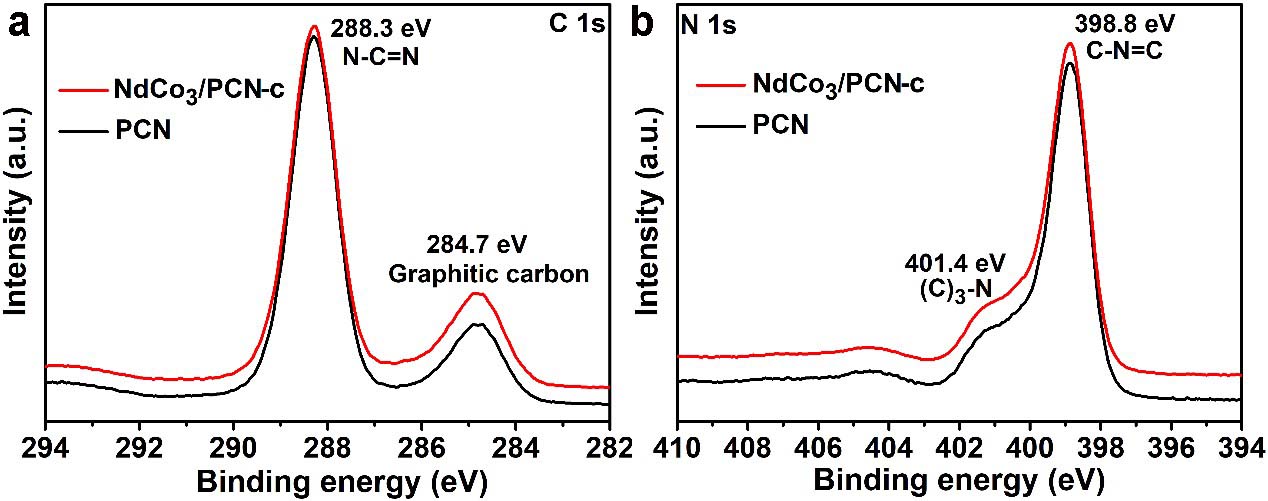


**Supplementary Figure 8 |** (a) C 1s XPS of PCN and NdCo_3_/PCN-c; (b) N 1s XPS of PCN and NdCo_3_/PCN-c.


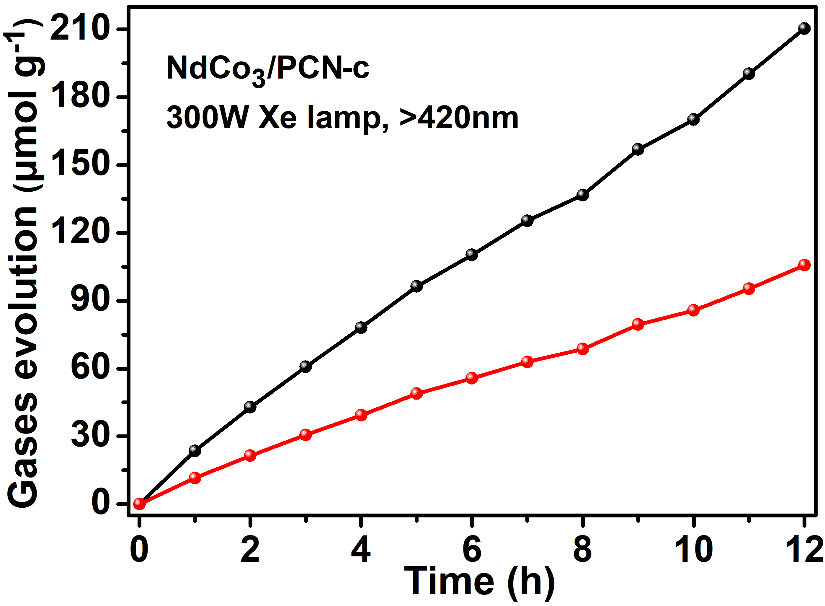


**Supplementary Figure 9 |** A time course of H_2_ and O_2_ production from water splitting under simulated sunlight (*λ*>420 nm) irradiation by NdCo_3_/PCN-c. Reaction conditions: photocatalyst, 40 mg; reactant solution, pure water (100 mL); light source, 300 W Xe lamp.


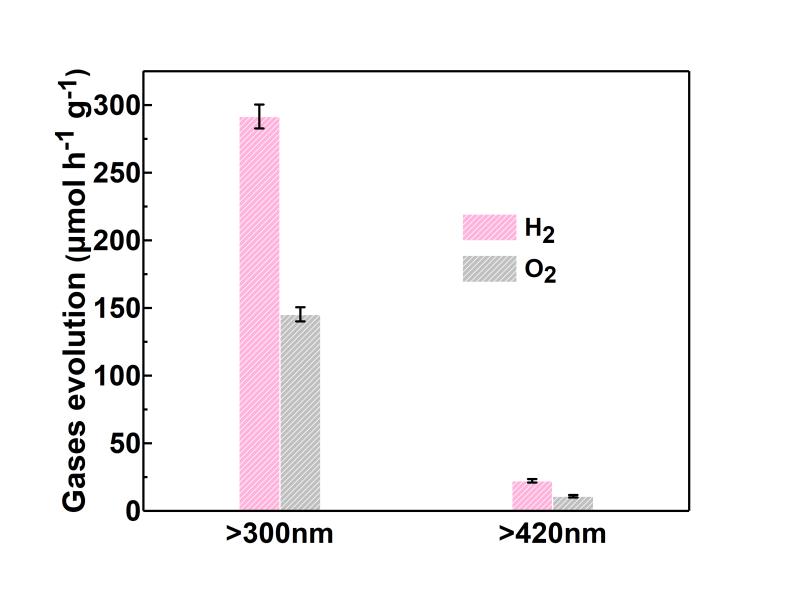


**Supplementary Figure 10 |** The gases evolutions of NdCo_3_/PCN-c simulated solar (*λ*>300 nm) irradiation and under simulated sunlight (*λ*>420 nm) irradiation.


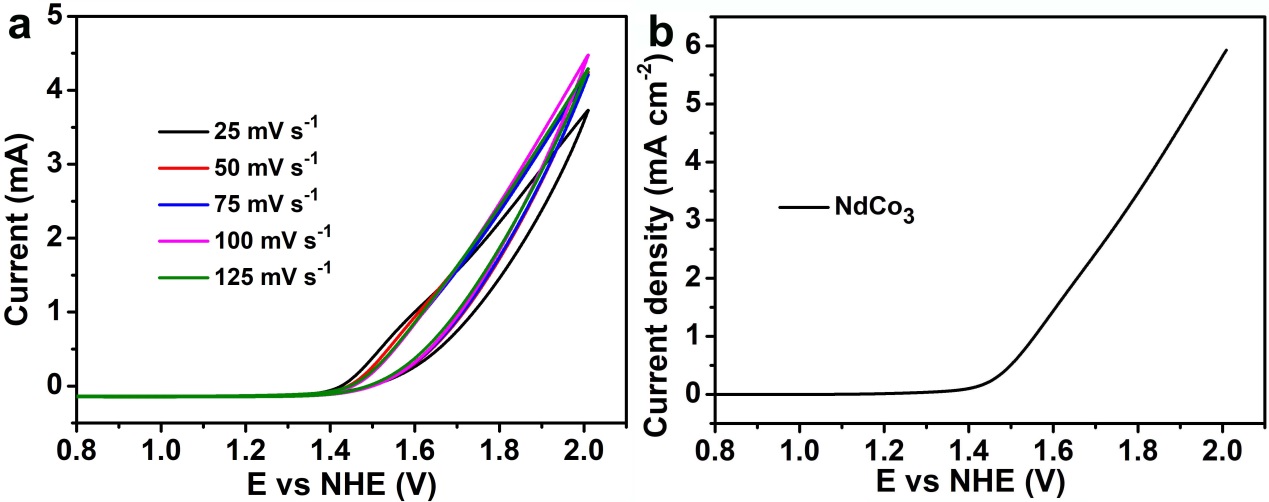


**Supplementary Figure 11 |** (a) The CV and (b) LSV of LnCo_3_ cluster in 0.5 M NaAc/HAc buffer solution (pH=6). The CV and LSV measurements were performed in the cell equipped with three electrodes, working electrode, counter electrode (Pt plate) and reference electrode (Ag/AgCl) in 0.5 M NaAc/HAc buffer solution (pH = 6). The LnCo_3_ cluster has obviously photo-catalytic water oxidation potency. The overpotential for NdCo_3_ to reach 1 mA cm^-2^ is 325 mV.


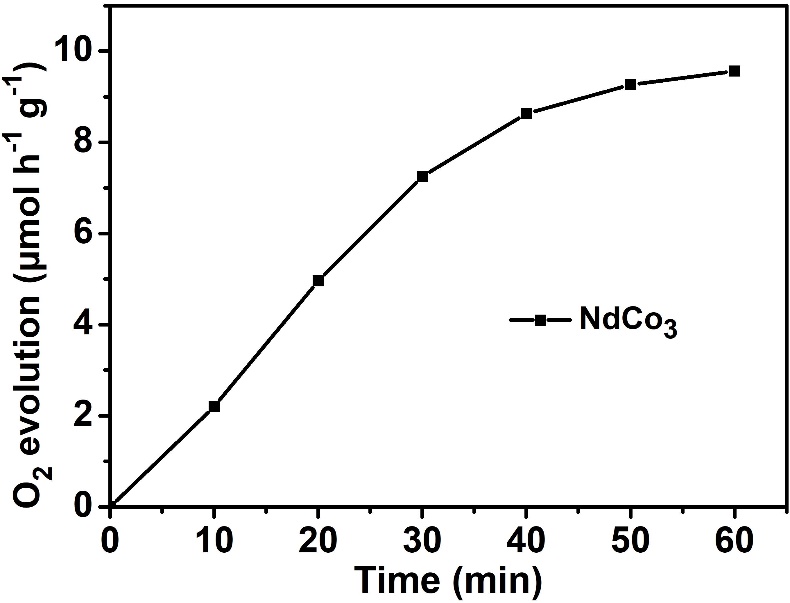


**Supplementary Figure 12 |** Visible-light-driven WOC activity of NdCo_3_ (1 mM [Ru(bpy)_3_]^2+^ as photosensitizer, 5 mM Na_2_S_2_O_8_ as sacrificial reagent) in 20 mL 0.5 M NaAc/HAc (pH=8) as buffer solution under λ≥420 nm light irradiation.


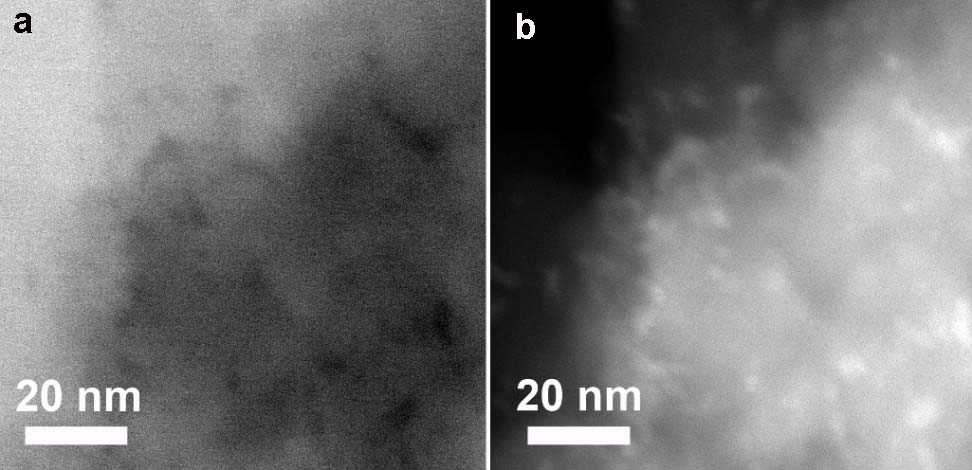


**Supplementary Figure 13 |** (a) TEM image and (b) HAADF-STEM image of NdCo_3_/PCN-c after photocatalysis.


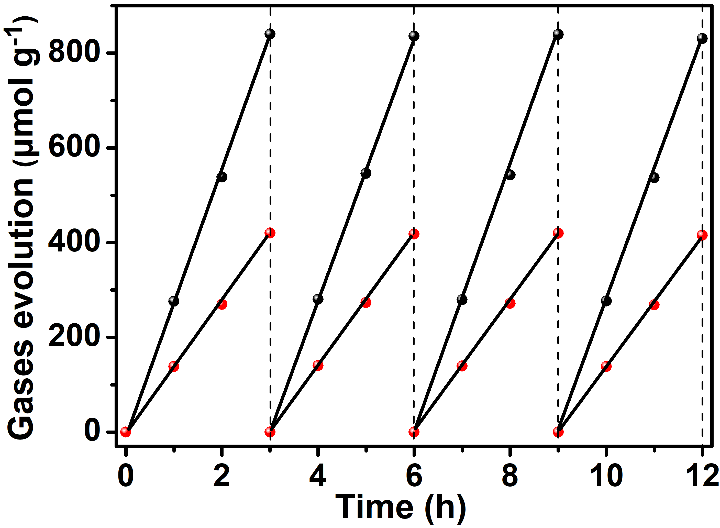


**Supplementary Figure 14 |** A time course of H_2_ and O_2_ production from water splitting under simulated sunlight (*λ*>300nm) irradiation of EuCo_3_/PCN. Reaction conditions: photocatalyst, 40 mg; reactant solution, pure water (100 mL); light source, 300 W Xe lamp.


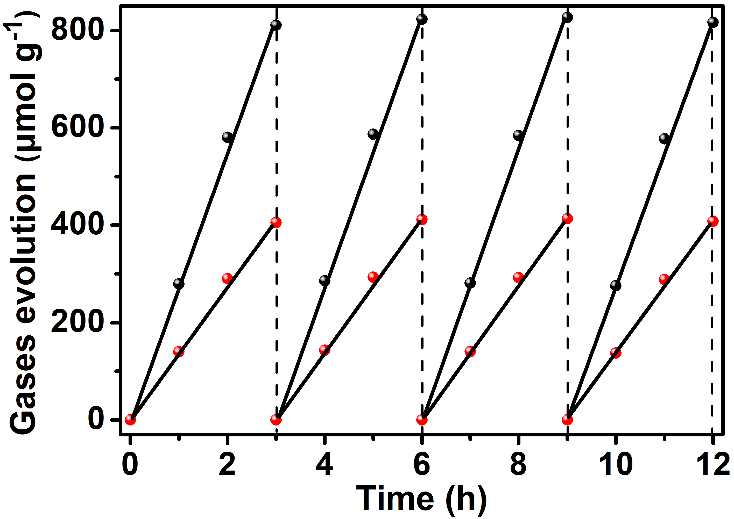


**Supplementary Figure 15 |** A time course of H_2_ and O_2_ production from water splitting under simulated sunlight (*λ*>300 nm) irradiation of CeCo_3_/PCN. Reaction conditions: photocatalyst, 40 mg; reactant solution, pure water (100 mL); light source, 300 W Xe lamp.


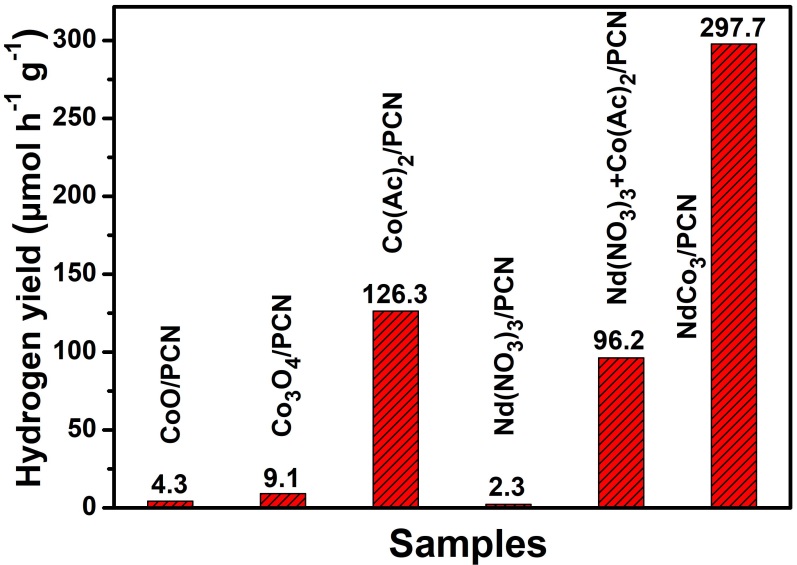


**Supplementary Figure 16 |** The comparison hydrogen yield of the Co(Ac)_2_/PCN, CoO/PCN, Co_3_O_4_/PCN, Nd(NO_3_)_3_/PCN, NdCo_3_/PCN-c and [Co(Ac)_2_+Nd(NO_3_)_3_]/PCN samples. Reaction conditions: photocatalyst, 40 mg; reactant solution, pure water (100 mL); light source, 300 W Xe lamp.


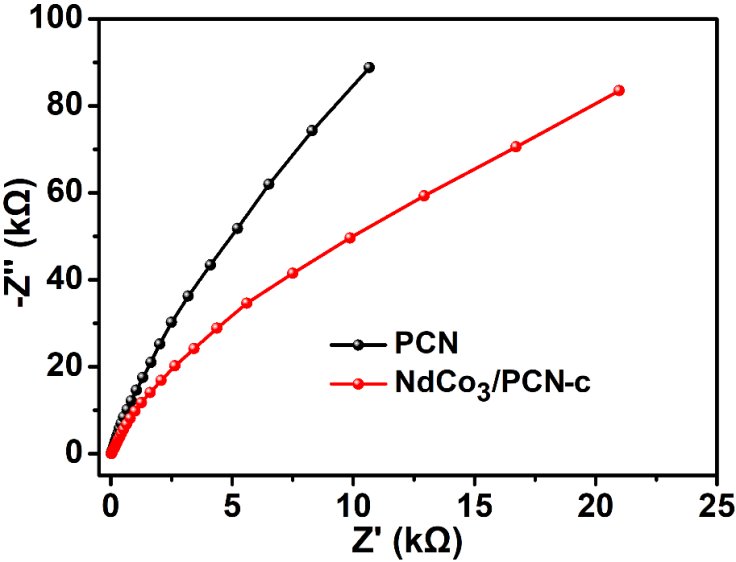


**Supplementary Figure 17 |** EIS of PCN and NdCo_3_/PCN-c in water.


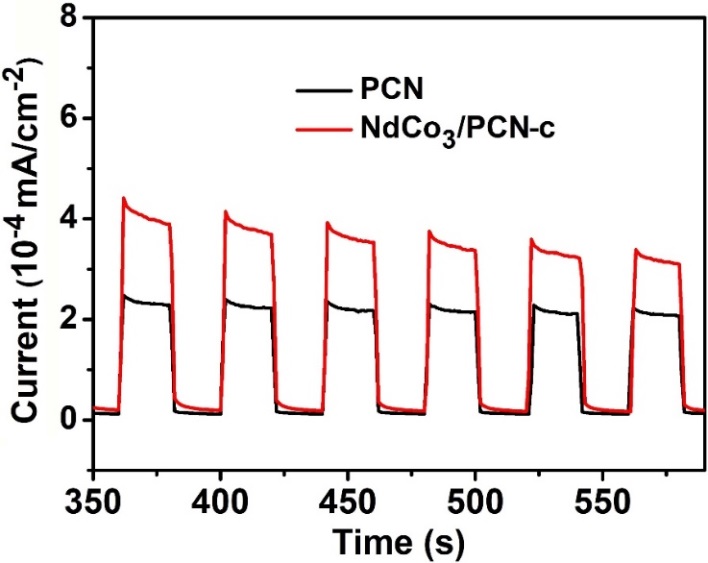


**Supplementary Figure 18 |** Transient photocurrent response of PCN and NdCo_3_/PCN-c in water under simulated irradiation at 0 V vs. Ag/AgCl (300 W Xe lamp).


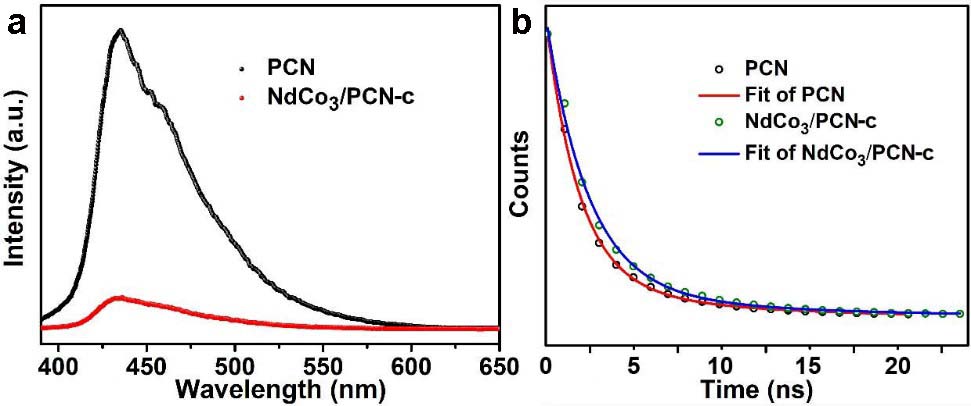


**Supplementary Figure 19 |** (a) PL spectra of PCN and NdCo_3_/PCN-c; (b) Time-resolved fluorescence spectra of PCN and NdCo_3_/PCN-c monitored at 430 nm under irradiation by a 368 nm laser at room temperature.


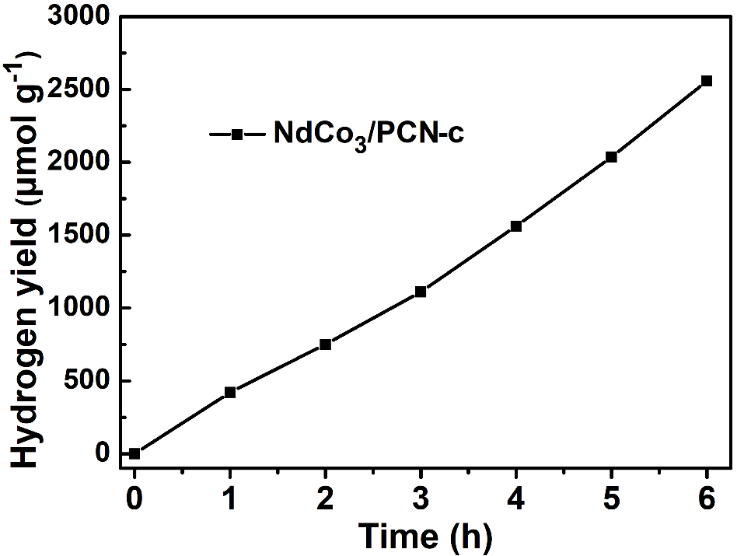


**Supplementary Figure 20 |** The photocatalytic H_2_ production performances of NdCo_3_/PCN-c in the presence of CH_3_OH.


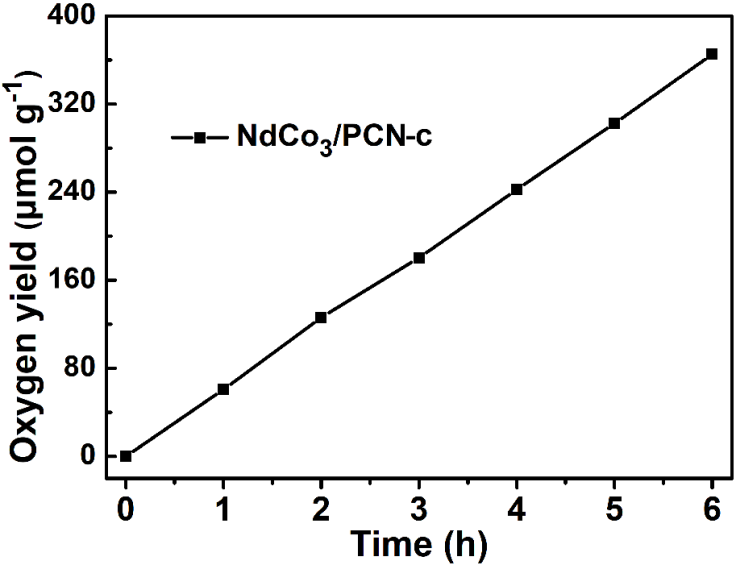


**Supplementary Figure 21 |** The photocatalytic O_2_ production performances of NdCo_3_/PCN-c in the presence of AgNO_3_.


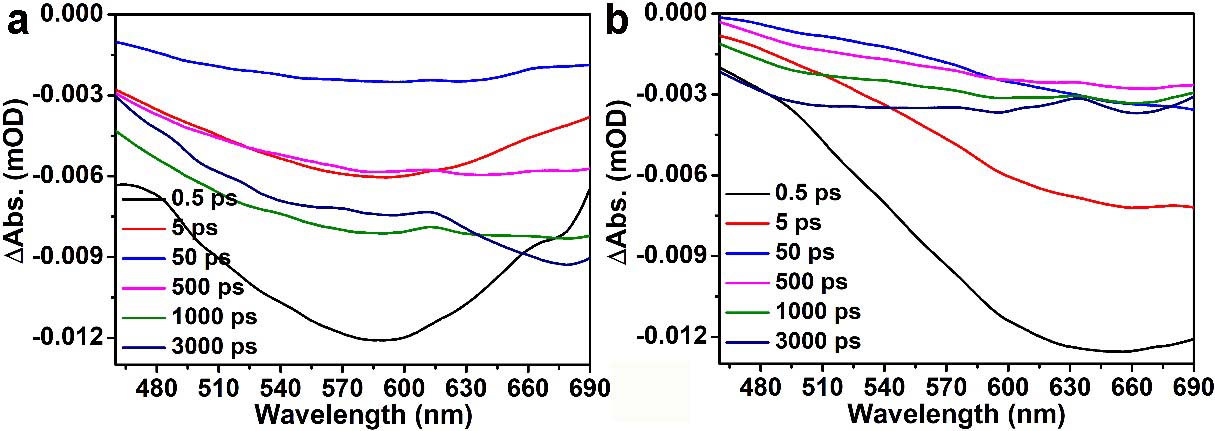


**Supplementary Figure 22 |** Representative TA spectra at different probe delays with (a) PCN and (b) NdCo_3_/PCN-c.


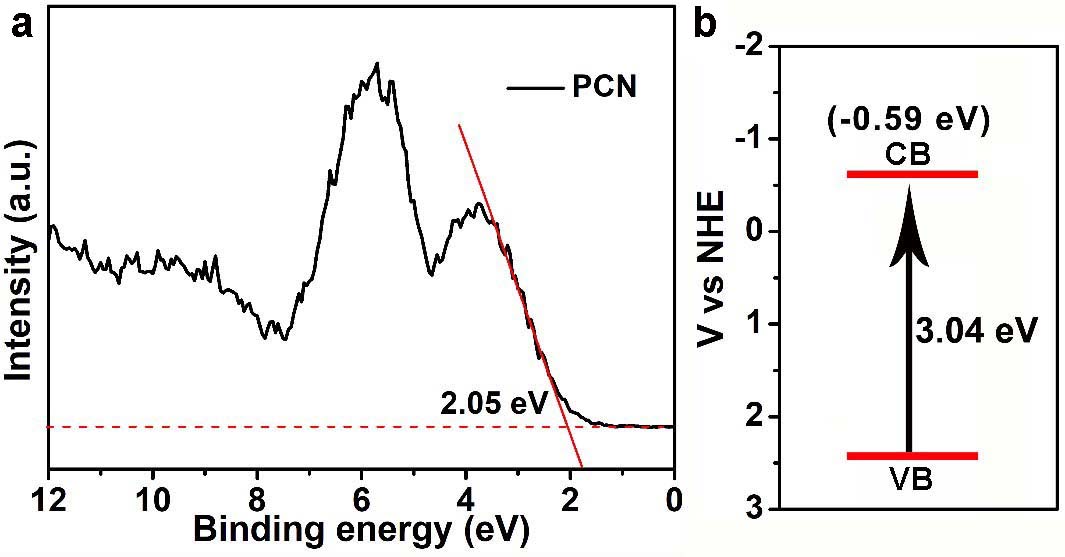


**Supplementary Figure 23 |** (a) XPS spectra of PCN; (b) CB and VB of PCN.

1. **Computational Details**

Spin polarized Density functional theory calculation were performed using VASP program package^[1]^. Perdew-Burke-Ernzerhof (PBE)^[2]^ functional was used to describe the exchange-correlation effect. The projector-augmented wave (PAW) approach^[3]^, featuring achieve simultaneously the computational efficiency of the pseudopotential method as well as the accuracy of the full-potential linearized augmented-plane wave (FLAPW) method, was used to treat the interaction between ions and electrons. Plane wave function with kinetic energy less than E_cut_ of 450 eV is included in the basic set. The K-point in the Brillouin zone was set to 2 × 2×1 grid^[4]^, having enough accuracy to calculate total energy. In order to get accurate adsorption between reaction species and catalyst, zero damping DFT-D3 method of Grimme^[5]^ was used to correct weak dispersion interaction. To account for the strong Coulomb repulsion of metal ions (e.g. Nd, Co) and reduce the self-interaction error from PBE functional, DFT+U^[6]^ strategy was used to correct the on-site Coulomb repulsions, where both *U*_eff_ value were obtained based on linear response strategy (see following part). According to magnetic measurement result (Supplementary Figure 24), NdCo_3_ cluster features antiferromagnetic property and the initial spin state of NdCo_3_/PCN were set to antiferromagnetic-spin distribution with high-spin distributions because of weak coordination field. For the most stable adsorption structure of reaction species, broking-symmetry calculations of Noodleman^[7]^ with different initial spin states were carried out.

**Computation of *U*_eff_ value:**

***U*_eff_** value is critical to accurately identify electronic structures of transition metal ions. Herein, the *U*_eff_ of Fe ions was identified by using the linear response approach introduced by Cococcioni et.al^[6]^. Perdew, Burke and Ernzerhof (PBE)^[2]^ functional was used to describe the exchange-correlation functional between electrons. Meanwhile, electronic wave functions were described by using vanderbilt ultrasoft pseudopotentials with the plane-wave energy cutoff of 45 Ryd. Convergence threshold for self-consistent calculation was set to 10^-9^. The relaxed NdCo_3_ cluster was loaded in the cell of 20×20×20 Å^3^. Thus, the 1×1×1 Monkhorst-Pack grid^[4]^ was enough to the sampling of Brillouin zone for cluster. After a careful convergence test, the small value of 0.001 eV Gaussian smearing was used to identify the partial occupancy of states.

In general, the total energy of DFT+U can be described as follow:


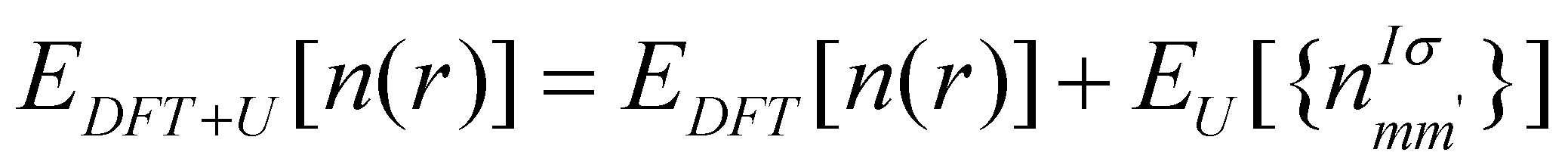
 **Eq(6)**

Where *E*_DFT_ represent a total energy from non-interaction Kohn-Sham algorithm; *E*_U_ is Hubbard correction energy.

Furthermore, if neglecting higher-multipolar terms of Coulomb interaction, *E*_U_ is written as:


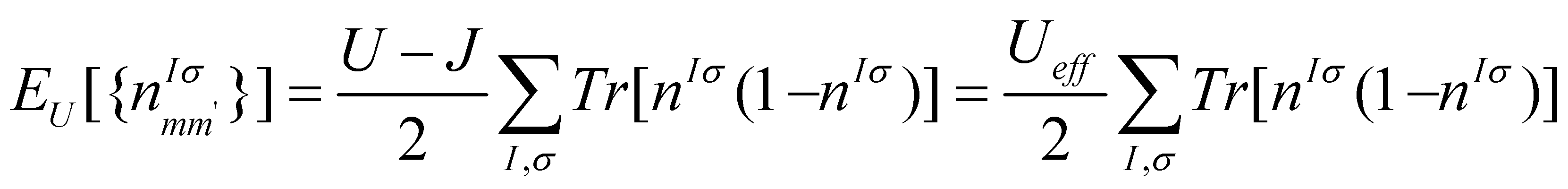
 **Eq(7)**

By linear-response approach U method, the linear response function χ is defined as

 **Eq(8)**

In this method, the interacting (χ) and non-interaction density response functions with respect to localized perturbations (typically from －0.1 eV to 0.1 eV) were firstly calculated. Thus *U*_eff_ can be obtained by following formula (see Eq(9)).

$U_{eff}=\frac{1}{{}_{0}}-\frac{1}{}$ **Eq(9)**

Via changing the rigid potential shifts α, the bare and self-consistent occupation regression response functions were achieved. Obviously, the interacting (χ) and the noninteracting (χ_0_) are the slopes of bare and self-consistent regression response functions (see Supplementary Figure 27), respectively. Therefore, the obtained U*_eff_* are 8.02 eV for Co ions and 6.09 eV for Nd ions. In this part, the calculations were performed using PWSCF program^[8]^ of ESPRESSO 6.1 software package in the supercomputer of TIAN-HE II, GuangZhou, China.


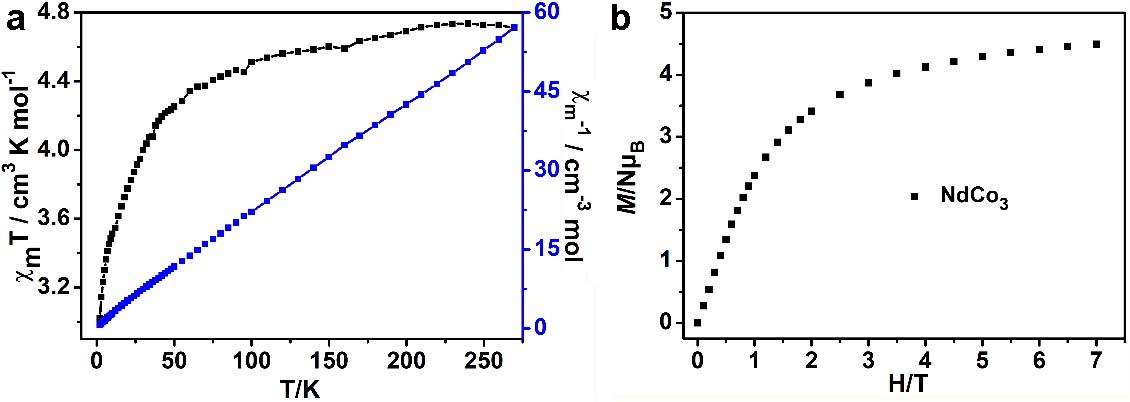


**Supplementary Figure 24 |** (a) Experiment plots of χ_m_T vs T of NdCo_3_ cluster; (b) Magnetization versus *H*/T for NdCo_3_ cluster.

**
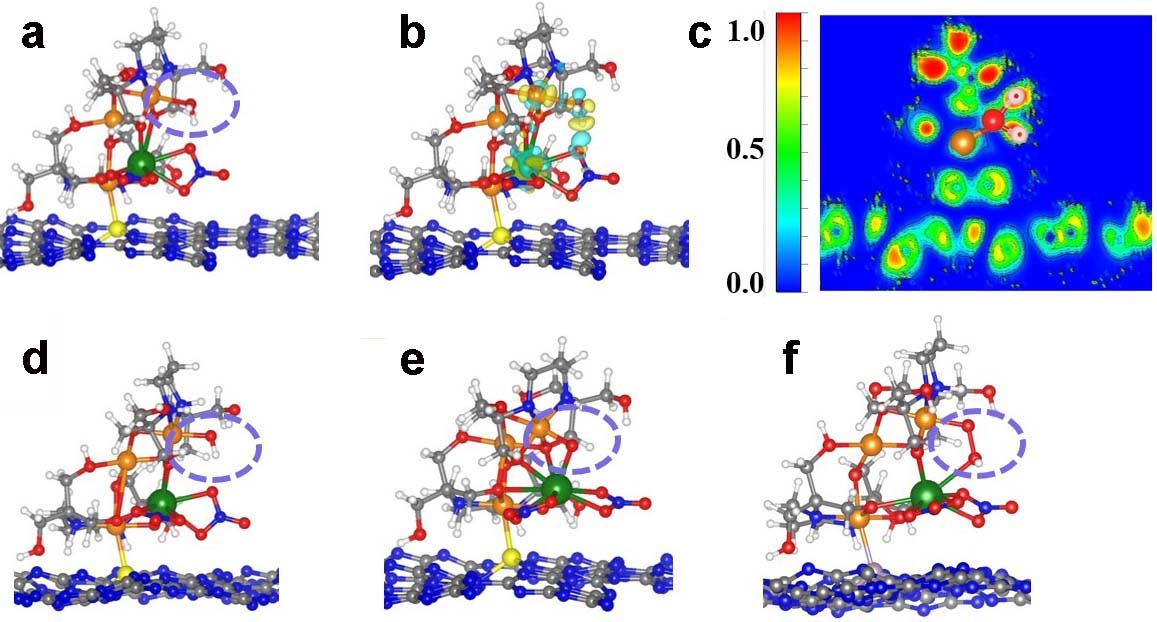
**

**Supplementary Figure 25 |** Adsorption structure (a), charge density difference (b) and Electron localization function (c) of H_2_O/NdCo_3_/PCN. Adsorption structure of OH/NdCo_3_/PCN-c (d), O/NdCo_3_/PCN-c (e) and OOH/NdCo_3_/PCN-c (f). Nd: green, Co: orange, O: red, N: blue, C: gray, P: yellow, H: white.


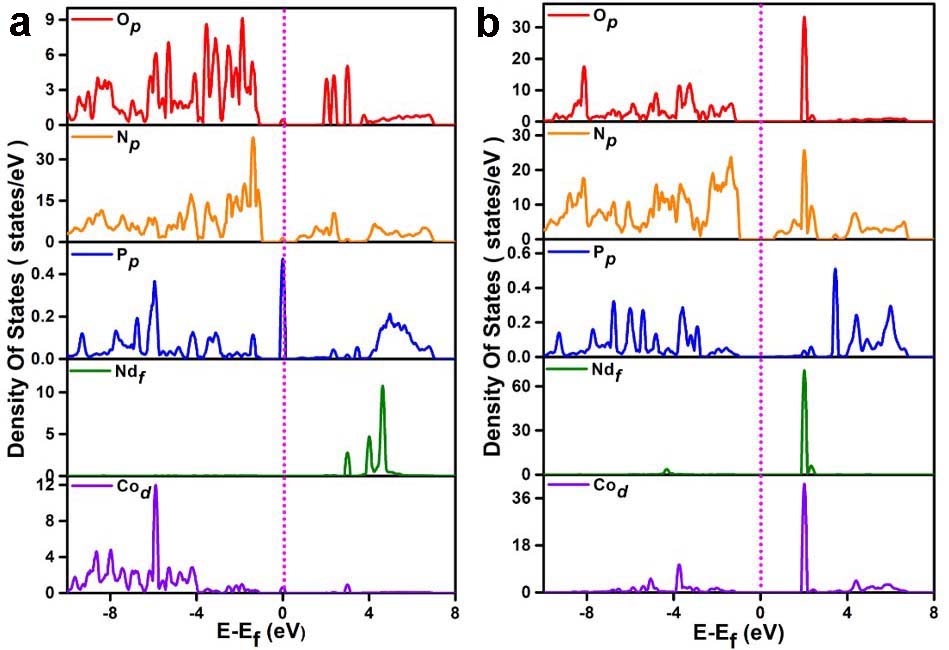


**Supplementary Figure 26 |** Partial density of states (spin up (a), spin down (b)) of H_2_O adsorption structure on the NdCo_3_/PCN-c (*E*_f_: energy of Fermi level).


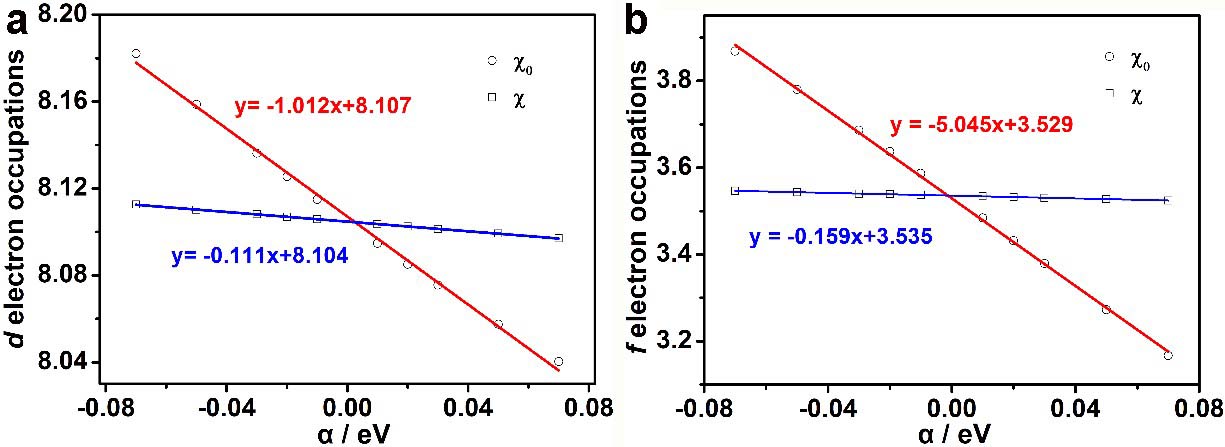


**Supplementary Figure 27 |** Relation curve of electronic occupations *vs* potential shifts from PWSCF software.

**
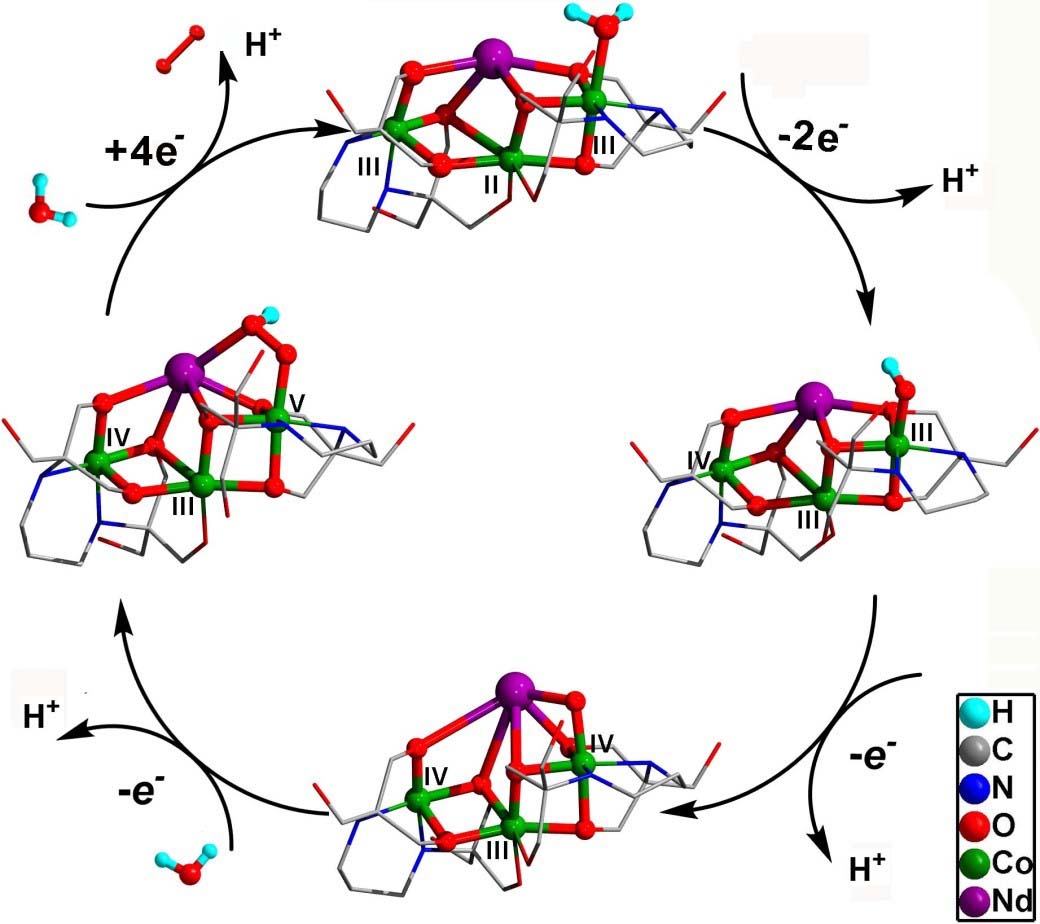
**

**Supplementary Figure 28 |** Proposed water oxidation reaction mechanism.

**Supplementary Table 4.** The fitting of the Time-resolved fluorescence spectra of PCN and NdCo_3_/PCN-c.

| Lifetime | | | | | | | |
| --- | --- | --- | --- | --- | --- | --- | --- |
| PCN | | | | NdCo_3_/PCN-c | | | |
| A1 | 7778 | τ1 | 1.85 | A1 | 8090 | τ1 | 2.40 |
| A2 | 1286 | τ2 | 8.03 | A2 | 9272 | τ2 | 11.2 |
| R-square | | 0.999 | | R-square | | 0.997 | |

**Supplementary Table 5.** Spin projection magnetization of selective atoms.

| Label^[1]^ | Bare | H_2_O-ad | OH-ad | O-ad | OOH-ad |
| --- | --- | --- | --- | --- | --- |
| P | 0.015 | 0.015 | 0.051 | 0.013 | -0.012 |
| Co(CUS) | 0.982 | 0.964 | 0.001 | 2.875 | -2.648 |
| Co(M) | 2.855 | -1.042 | 0.322 | -0.008 | -0.656 |
| Co(P) | 0.944 | 2.821 | -0.687 | 2.422 | 2.720 |
| Nd | -1.001 | -0.996 | -3.005 | -1.004 | 1.032 |

**^[1]^Co(CUS):** cobalt ion with coordination unsaturated site; **Co(M):** middle cobalt ion; **Co(P):** cobalt ion linking with P atom in **PCN**

**Supplementary Table 6.** Summary of the mass ratios of NdCo_3_ cluster to PCN, H_2_ production rates of the prepared samples (40 mg catalysts, λ > 300 nm).

| Samples | Nd (atom) | Co (atom) | Mass ratio (wt%) | H_2_ production rate (μmol h^-1^ g^-1^) |
| --- | --- | --- | --- | --- |
| NdCo_3_/PCN-a | 2.5 | 6.7 | 0.36 | 41.3 |
| NdCo_3_/PCN-b | 4.2 | 13.1 | 0.61 | 207.1 |
| NdCo_3_/PCN-c | 7.3 | 22.3 | 1.05 | 297.7 |
| NdCo_3_/PCN-d | 10.8 | 32.6 | 1.55 | 241.6 |
| NdCo_3_/PCN-e | 14.1 | 43.6 | 2.03 | 237.4 |

**Supplementary Table 7.** Summary of the mass ratios of LnCo_3_ cluster to PCN, H_2_ production rates of the prepared samples (40 mg catalysts, λ > 300 nm).

| Samples | Eu (atom) | Ce (atom) | Co (atom) | Mass ratio (wt%) | H_2_ production rate (μmol h^-1^ g^-1^) |
| --- | --- | --- | --- | --- | --- |
| EuCo_3_/PCN | 6.7 | **_** | 18.8 | 0.97 | 279.1 |
| CeCo_3_/PCN | **_** | 8.2 | 24.7 | 1.18 | 274.5 |

**Supplementary Table 8.** Selected Bond distances (Å) of compound **1**.

| **Bond** | **Dis(Å)** | **Bond** | **Dis(Å)** |
| --- | --- | --- | --- |
| Nd1-O9 | 2.441(3) | Co1-O14 | 1.929(4) |
| Nd1-O1 | 2.443(3) | Co1-N2 | 1.964(5) |
| Nd1-O13 | 2.456(4) | Co2-O9 | 1.871(4) |
| Nd1-O12 | 2.480(3) | Co2-O8 | 1.884(4) |
| Nd1-O4 | 2.482(3) | Co2-O12 | 1.915(3) |
| Nd1-O16 | 2.485(4) | Co2-O15 | 1.928(4) |
| Nd1-O20 | 2.565(3) | Co2-N3 | 1.950(5) |
| Nd1-O24 | 2.589(4) | Co2-N4 | 1.966(5) |
| Nd1-O23 | 2.699(4) | Co3-O8 | 1.989(4) |
| Nd1-O21 | 2.736(4) | Co3-O3 | 2.013(4) |
| Co1-O1 | 1.880(3) | Co3-O11 | 2.113(4) |
| Co1-O3 | 1.884(3) | Co3-O6 | 2.138(4) |
| Co1-O4 | 1.908(4) | Co3-O12 | 2.147(3) |
| Co1-N1 | 1.922(5) | Co3-O4 | 2.147(3) |
| ^1^1-X,-Y,-Z; ^2^-X,1-Y,-Z | | | |

**Supplementary Table 9.** Selected Bond distances (Å) of compound **2**.

| **Bond** | **Dis(Å)** | **Bond** | **Dis(Å)** |
| --- | --- | --- | --- |
| Eu1-O9 | 2.409(5) | Co1- O14 | 1.925(5) |
| Eu1-O13 | 2.421(6) | Co1-N1 | 1.946(7) |
| Eu1-O1 | 2.425(5) | Co1-N2 | 1.953(6) |
| Eu1-O12 | 2.442(5) | Co2-O9 | 1.876(5) |
| Eu1-O16 | 2.444(6) | Co2-O8 | 1.885(5) |
| Eu1-O4 | 2.454(5) | Co2-O12 | 1.907(5) |
| Eu1-O20 | 2.525(5) | Co2-O15 | 1.916(6) |
| Eu1-O24 | 2.544(5) | Co2-N3 | 1.931(7) |
| Eu1-O23 | 2.702(6) | Co2-N4 | 1.971(7) |
| Eu1-O21 | 2.798(6) | Co3-O8 | 1.988(6) |
| Eu1-N5 | 3.056(7) | Co3-O3 | 2.016(5) |
| Eu1-N6 | 3.065(7) | Co3-O11 | 2.115(5) |
| Co1-O1 | 1.866(5) | Co3-O6 | 2.143(5) |
| Co1-O3 | 1.897(5) | Co3-O12 | 2.148(5) |
| Co1-O4 | 1.905(5) | Co3-O4 | 2.153(5) |
| ^1^1-X,-Y,-Z; ^2^-X,1-Y,-Z | | | |

**Supplementary Table 10.** Selected Bond distances (Å) of compound **3**.

| **Bond** | **Dis(Å)** | **Bond** | **Dis(Å)** |
| --- | --- | --- | --- |
| Ce1-O3 | 2.443(6) | Co1-O5 | 1.899(6) |
| Ce1-O7 | 2.494(7) | Co1-O8 | 1.926(7) |
| Ce1-O5 | 2.506(5) | Co1-N2 | 1.949(8) |
| Ce1-O11 | 2.588(6) | Co1-N1 | 1.949(8) |
| Ce1-O10 | 2.738(8) | Co2-O2 | 1.984(6) |
| Co1-O2 | 1.878(6) | Co2-O4 | 2.109(6) |
| Co1-O3 | 1.889(6) | Co2-O5 | 2.144(6) |
| ^1^3/2-X,+Y,3/2-Z | | | |

**Supplementary Table 11.** The calculated states of the metal ions.

| Atom | Atom | Distance | BVS |
| --- | --- | --- | --- |
| Co3 | O6 | 1.885 | 0.511 |
|  | O8 | 1.907 | 0.482 |
|  | O5 | 1.879 | 0.520 |
|  | O4 | 1.927 | 0.457 |
|  | N5 | 1.966 | 0.558 |
|  | N6 | 1.923 | 0.626 |
| Total |  | | 3.154 |
| Co2 | O6 | 2.012 | 0.363 |
|  | O8 | 2.145 | 0.253 |
|  | O14 | 2.147 | 0.252 |
|  | O15 | 2.112 | 0.277 |
|  | O9 | 2.134 | 0.261 |
|  | O11 | 1.991 | 0.384 |
| Total |  | | 1.79 |
| Co1 | O2 | 1.927 | 0.457 |
|  | O12 | 1.874 | 0.527 |
|  | O14 | 1.914 | 0.473 |
|  | O11 | 1.882 | 0.516 |
|  | N3 | 1.953 | 0.578 |
|  | N4 | 1.965 | 0.560 |
| Total |  | | 3.111 |

**Supplementary Table 12.**  The calculated states of two nitrogen and six oxygen atoms in the ligand.

| Atom | Atom | Distance | BVS |
| --- | --- | --- | --- |
| N5 | C7 | 1.486 | 0.888 |
|  | C8 | 1.505 | 0.843 |
|  | Co3 | 1.966 | 0.411 |
| Total |  |  | 2.142 |
| N6 | C5 | 1.489 | 0.881 |
|  | C2 | 1.512 | 0.828 |
|  | Co3 | 1.923 | 0.462 |
| Total |  |  | 2.171 |
| O6 | C4 | 1.437 | 0.881 |
|  | Co2 | 2.012 | 0.363 |
|  | Co3 | 1.855 | 0.555 |
| Total |  |  | 1.799 |
| O9 | Co2 | 2.134 | 0.297 |
|  | C9 | 1.441 | 0.871 |
| Total |  |  | 1.168 |
| O5 | C3 | 1.413 | 0.940 |
|  | Co3 | 1.879 | 0.520 |
|  | Nd1 | 2.448 | 0.376 |
| Total |  |  | 1.836 |
| O8 | Nd1 | 2.483 | 0.342 |
|  | Co3 | 1.907 | 0.482 |
|  | Co2 | 2.145 | 0.288 |
|  | C11 | 1.428 | 0.902 |
| Total |  |  | 2.014 |
| O7 | C1 | 1.425 | 0.910 |
| O10 | C10 | 1.430 | 0.898 |

**Supplemental References**

[1] Kresse G, Hafner J. Ab initio molecular dynamics for liquid metals. J. *Phys. Rev. B* 1993; **47**: 558-561.

[2] (a) Perdew J P, Burke K, Ernzerhof M. Generalized Gradient Approximation Made Simple. *Phys. Rev. Lett.* 1997; **78**: 1396-1396; (b) Blöchl P E. Projector augmented-wave method. *Phys. Rev. B* 1994; **50**: 17953-17979.

[3] Kresse G, Furthmüller J. Efficiency of ab-initio total energy calculations for metals and semiconductors using a plane-wave basis set. *Comput. Mater. Sci.* 1996; **6**: 15-50.

[4] Monkhorst H J, Pack J D. Special points for Brillouin-zone integrations. *Phys. Rev. B* 1976; **13**: 5188-5192.

[5] Grimme S, Semiempirical GGA‐type density functional constructed with a long‐range dispersion correction. *J. Comput. Chem. Phys.* 2006; **27**: 1787-1799.

[6] Cococcioni M, de Gironcoli S. Linear response approach to the calculation of the effective interaction parameters in the LDA+U method. *Phys. Rev. B* 2005; **71**: 035105.

[7] (a) Noodleman L, Norman J G. The Xα valence bond theory of weak electronic coupling. Application to the low‐lying states of Mo_2_Cl_8_^4−^. *J. Chem. Phys.* 1979; **70**: 4903-4906; (b) Noodleman L. Valence bond description of antiferromagnetic coupling in transition metal dimers. *J. Chem. Phys.* 1981; **74**: 5737-5743.
